# Insights into the Evolution of the New World Diploid Cottons (*Gossypium*, Subgenus *Houzingenia*) Based on Genome Sequencing

**DOI:** 10.1093/gbe/evy256

**Published:** 2018-11-23

**Authors:** Corrinne E Grover, Mark A Arick, Adam Thrash, Justin L Conover, William S Sanders, Daniel G Peterson, James E Frelichowski, Jodi A Scheffler, Brian E Scheffler, Jonathan F Wendel

**Affiliations:** 1 Department of Ecology, Evolution, and Organismal Biology, Iowa State University; 2 Institute for Genomics, Biocomputing, and Biotechnology, Mississippi State University; 3 Department of Computer Science & Engineering, Mississippi State University; 4 The Jackson Laboratory, Connecticut; 5 USDA, Crop Germplasm Research Unit, College Station, Texas; 6 USDA, Crop Genetics Research Unit, Stoneville, Mississippi; 7 USDA, Genomics and Bioinformatics Research Unit, Stoneville, Mississippi

**Keywords:** phylogenomics, molecular evolution, transposable elements, hybridization, introgression, rate variation

## Abstract

We employed phylogenomic methods to study molecular evolutionary processes and phylogeny in the geographically widely dispersed New World diploid cottons (*Gossypium*, subg. *Houzingenia*). Whole genome resequencing data (average of 33× genomic coverage) were generated to reassess the phylogenetic history of the subgenus and provide a temporal framework for its diversification. Phylogenetic analyses indicate that the subgenus likely originated following transoceanic dispersal from Africa about 6.6 Ma, but that nearly all of the biodiversity evolved following rapid diversification in the mid-Pleistocene (0.5–2.0 Ma), with multiple long-distance dispersals required to account for range expansion to Arizona, the Galapagos Islands, and Peru. Comparative analyses of cpDNAversus nuclear data indicate that this history was accompanied by several clear cases of interspecific introgression. Repetitive DNAs contribute roughly half of the total 880 Mb genome, but most transposable element families are relatively old and stable among species. In the genic fraction, pairwise synonymous mutation rates average 1% per Myr, with nonsynonymous changes being about seven times less frequent. Over 1.1 million indels were detected and phylogenetically polarized, revealing a 2-fold bias toward deletions over small insertions. We suggest that this genome down-sizing bias counteracts genome size growth by TE amplification and insertions, and helps explain the relatively small genomes that are restricted to this subgenus. Compared with the rate of nucleotide substitution, the rate of indel occurrence is much lower averaging about 17 nucleotide substitutions per indel event.

## Introduction

**Fig. 1.**
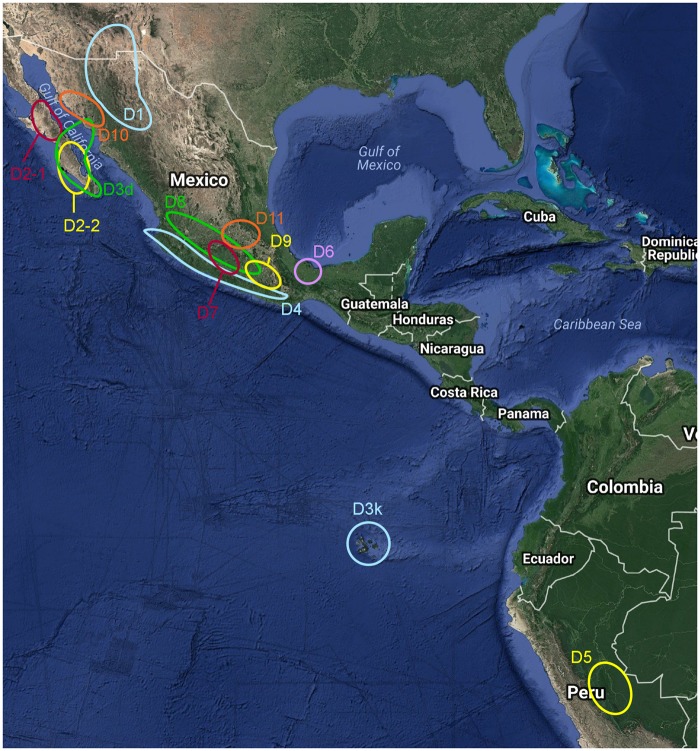
—Approximate geographic ranges of *Houzingenia* species. D1 = *G. thurberi*, D2-1 = *G. armourianum*, D2-2 = *G. harknessii*, D3d = *G. davidsonii*, D3k = *G. klotzschianum*, D4 = *G. aridum*, D5 = *G. raimondii*, D6 = *G. gossypioides*, D7 = *G. lobatum*, D8 = *G. trilobum*, D9 = *G. laxum*, D10 = *G. turneri*, and D11 = *G. schwendimanii*.

The American, diploid “D-genome” cottons (subgenus *Houzingenia*) comprise a monophyletic clade of cytogenetically and morphologically distinct species largely distributed from Southwest Mexico to Arizona, with additional disjunct species distributions in Peru and the Galapagos Islands (Fryxell 1979; Endrizzi et al. 1985; Álvarez et al. 2005; Wendel and Grover 2015) (fig. 1). Included in the 13–14 species presently recognized in subgenus *Houzingenia* (Ulloa et al. 2013; Wendel and Grover 2015) is a source of cytoplasmic male sterility in cotton, *Gossypiumharknessii* Brandegee, as well as the model diploid, D-genome progenitor to wild and domesticated allopolyploid (AD-genome) cotton, *Gossypiumraimondii* Ulbrich [reviewed in {Wendel and Grover 2015}]. The close relationship of *Houzingenia* species to the agronomically important polyploid cottons has stimulated considerable interest in their diversity, distribution, and phylogenetic relationships. Accordingly, many of the species in the subgenus are taxonomically well-understood, although their phylogenetic relationships remain incompletely resolved.

Early taxonomists divided subgenus *Houzingenia* into two sections and six subsections. These species alignments have, for the most part, been reiterated in subsequent phylogenetic studies (Wendel and Albert 1992; Wendel et al. 1995; Cronn et al. 1996; Seelanan et al. 1997; Small and Wendel 2000; Álvarez et al. 2005), at least at the subsectional level. The alignment of subsections into their present taxonomic circumscriptions, however, does not appear to represent natural clades. Several molecular data sets have been used to evaluate these relationships, including chloroplast restriction sites (Wendel and Albert 1992); simple sequence repeat (SSR) and expressed sequence tag (EST)–SSR markers (Guo et al. 2007; Zhu et al. 2009); random amplified polymorphic DNA (RAPD) markers (Khan et al. 2000); internal transcribed sequences (ITS) (Álvarez et al. 2005); and a few single-copy nuclear genes (Álvarez et al. 2005). Relationships among the six subsections remain unclear, with different studies yielding alternative topologies (Cronn et al. 1996; Small and Wendel 2000; Liu and Wendel 2001; Álvarez et al. 2005); however, early morphological and cytogenetic comparisons using intergenomic hybrids have firmly established *G. raimondii* as the closest living relative to the D-genome ancestor of polyploid cotton species [reviewed in {Wendel and Cronn 2003}]. Subsequent analyses have supported this observation (Cronn et al. 1996, 1999; Seelanan et al. 1997; Small et al. 1998; Small and Wendel 2000; Abdalla et al. 2001; Liu and Wendel 2001) with few conflicts [however, see {Wendel et al. 1995}], as reviewed in Wendel and Grover (2015).

One consequence of these many molecular investigations has been the discovery of instances of putative hybridization among the D-genome cottons (Cronn and Wendel 2003), and, in one remarkable case (i.e., *Gossypiumgossypioides*), between a *Houzingenia* species and another, geographically isolated subgenus from Africa [either A-, B-, E-, or F-genome {Wendel et al. 1995; Cronn et al. 2003; Cronn and Wendel 2003}]. Most remarkably, *G. gossypioides* appears to have been introgressed multiple times, with an early nuclear introgression event followed by a much later hybridization to a member of the *G. raimondii* lineage, resulting in chloroplast, if not further (and cryptic), nuclear introgression (Cronn et al. 2003). Cytoplasmic introgression, and possibly cryptic nuclear introgression, is also present in some populations of *Gossypiumaridum*;that is, the Mexican Colima populations of *G. aridum* possess a *Gossypiumdavidsonii*- or *Gossypiumklotzschianum*-like cytoplasm (Álvarez et al. 2005).

Early attempts at understanding the evolution of the repetitive fraction of the genus support the inference of African introgression in *G. gossypioides* (Zhao et al. 1998); however, little else is understood with respect to the evolution of the nongenic fraction of *Houzingenia*. The D-genome cottons possess the smallest nuclear genomes in the genus, ranging only ∼1.11 fold, from 841 Mb to 934 Mb (Hendrix and Stewart 2005). Notably, the distribution of genome sizes among the subsections suggests that the subgenus has experienced differential growth and/or reduction in genome size among species; however, the sequences gained and/or lost have not been characterized. While the differences in genome size are not dramatic, the transposable element (TE) types that have accumulated in *G. raimondii* are different from those that have achieved higher copy numbers in the remainder of the genus (Hawkins et al. 2006; Renny-Byfield et al. 2016; Grover et al. 2017). Furthermore, research comparing the two sister genera to cotton [i.e., *Kokia* and *Gossypioides*; {Grover et al. 2017}] reveals that their equivalent genome sizes belies a more dynamic scenario of repetitive sequence gain and loss. A similar conclusion was reached for the two A-genome (subgenus *Gossypium*) species, whose small change in genome size (∼1.05×) masks differences in TE accumulation (Renny-Byfield et al. 2016; Grover et al. 2017).

Here, we re-examine phylogenetic relationships and molecular evolution in the cotton subgenus *Houzingenia* using next-gen (Illumina) sequencing data. We leverage newly generated genome and plastome sequences, the first for most of the included species, to address questions surrounding genome evolution in a monophyletic group of closely related species. We characterize both the pace and patterns of molecular evolution of genes and repetitive sequences, evaluate the amount of divergence outside of genes, and describe the history of indels and single-nucleotide polymorphisms (SNPs). Finally, we revisit the phylogeny of the D-genome clade, providing insight into relationships among species and with respect to sequence gain and loss among closely related species. Our results represent a phylogenomic characterization of molecular evolution for a closely related set of plant species and provide resources for comparative research and for the cotton community at large.

## Materials and Methods

### Sequence Generation and Initial Processing

DNA was extracted from leaves using either 1) a modified version of the method described by Dabo et al. (1993), or 2) the Qiagen DNeasy Plant Mini Kit (69104) followed by the DNeasy PowerClean Pro Cleanup kit (12997). For those accessions with sufficient DNA available from USDA-ARS, Stoneville MS (supplementary table 3, Supplementary Material online, BGI) samples were submitted to BGI Genomics (Hong Kong) for Illumina library preparation and 2 × 100 bp sequencing. For accessions with limited amounts of available DNA (supplementary table 3, Supplementary Material online, NXT), Illumina sequencing libraries were prepared in-house at the USDA-ARS GBRU core facility by the Nextera DNA Library Prep Kit (product number FC-121-1030 with adapter set FC-121-1011, Illumina, San Diego, CA, USA) according to the manufacturer’s protocol. Samples obtained from Iowa State University, Ames, IA (supplementary table 3, Supplementary Material online, USDA) were prepared at the USDA-ARS GBRU core facility using Accel-NGS 2S PCR-Free (Product number 20024 with adapter set 26396, Swift Biosciences, Ann Arbor, MI, USA). Library sizes were validated on the Agilent TapeStation 2200 High Sensitivity D1000 Assay (Part No. 5067-5584, Agilent Technologies, Santa Clara, CA, USA) and assayed for concentration prior to equimolar pooling by a KAPA Library Quantification Kit (Product number KK4854, Kapa Biosystems, Inc., Wilmington, MA, USA) on a qPCR instrument (LightCycler 96, Roche Applied Science, Indianapolis, IN, USA). Each pool was clustered onboard an Illumina HiSeq2500 DNA sequencer with a HiSeq PE (paired-end) Rapid v2 flowcell clustering kit (Product number PE-402-4002, Illumina, San Diego, CA, USA) and sequenced as 2 × 100 bp with the HiSeq Rapid SBS Kit v2 (Product number FC-402-4021, Illumina, San Diego, CA, USA). The remaining samples (supplementary table 3, Supplementary Material online, Novogene) were submitted to Novogene (Beijing) for Illumina library preparation and 2 × 150 bp sequencing. Reads are available from the Short-Read Archive (SRA) under PRJNA488266. The outgroup, *Gossypiumlongicalyx*, was downloaded from SRA (SRX204849) and processed alongside the *Houzingenia* samples.

Reads were trimmed and filtered with Trimmomatic v0.32 (Bolger et al. 2014) with the following options: 1) sequence adapter removal, 2) removal of leading and/or trailing bases when the quality score (*Q*) <28, 3) removal of bases after average *Q* < 28 (8 nt window) or single base quality <10, and 4) removal of reads <85 nt. Detailed parameters can be found at https://github.com/IGBB/D_Cottons_USDA, last accessed December 18, 2018.

### Genome Assembly and Annotation

Trimmed data were independently assembled for each species via ABySS v2.0.1 (Simpson et al. 2009), using every 5th kmer value from 40 through 100. A single assembly with the highest E-size (Salzberg et al. 2012) was selected for each species and subsequently annotated with MAKER v2.31.6 (Holt and Yandell 2011) using evidence from: 1) the NCBI *G. raimondii* EST database (Udall et al. 2006), 2) *G. raimondii* reference genome predicted proteins, as hosted by CottonGen.org (Paterson et al. 2012), and 3) three ab initio gene prediction programs, that is Genemark v4.30 (Borodovsky et al. 2003), SNAP v2013-11-29 (Korf 2004), and Augustus v3.0.3 (Stanke et al. 2006). Both the SNAP and Augustus models were trained using BUSCO v2.0 (Simão et al. 2015). Chromosomer version 0.1.3 (Tamazian et al. 2016), a reference-assisted scaffolder, was used to scaffold the selected assemblies against the gold standard *G. raimondii* genome. MAKER v2.31.6 (Holt and Yandell 2011) was used to transfer the previous annotations to the Chromosomer-based scaffolds by rerunning MAKER and using the transcripts from the original annotation as evidence. Assemblies are also available under PRJNA488266.

#### Phylogenetic Analyses and Ancestral State Reconstruction

Trimmed reads from the genome assembly were mapped against the *G. raimondii* reference sequence (Paterson et al. 2012) using BWA v0.7.10 (Li and Durbin 2009), postprocessed with samtools (Li et al. 2009), and individual genes were independently assembled for each species/accession via BamBam v1.3 (Page et al. 2013) in conjunction with the *G. raimondii* reference annotation (Paterson et al. 2012). Alignments were pruned for genes and/or alignment positions with insufficient coverage, that is, too many ambiguous bases, using filter_alignments (https://github.com/IGBB/D_Cottons_USDA/; last accessed December 18, 2018). Parameters were set to remove sequences with more than 10% ambiguous bases within species and to remove aligned positions with more than 10% ambiguity among species. Genes were additionally filtered by length, to retain only those genes between a minimum of 500 bp and a maximum of 4,051 bp, the latter of which represents the *G. raimondii* genome-wide mean plus three standard deviations. Only those genes with a minimum of one accession per species were retained for phylogenetic and molecular analyses. Genes were concatenated and subjected to maximum likelihood (ML) analysis via RaxML (Stamatakis 2014) using the basic general time reversible model with gamma distribution (GTRGAMMA), 10,000 alternative runs on distinct starting trees, and rapid bootstrapping with consensus tree generation. The ML trees were rooted with a member of subgenus *Longiloba*, *G. longicalyx* (African F-genome).

Molecular evolutionary analyses were conducted in R v3.4.4 (R Core Team 2018). Species divergence time estimates were calculated via chronos from {ape} (Paradis et al. 2004), using the divergence estimates previously calculated for the Malvaceae (Grover et al. 2017) and penalized likelihood (Sanderson 2002; Kim and Sanderson 2008) and maximum likelihood. Minimum and maximum node ages were specified for both the root and the node that separates *Erioxylum* from the rest of the subgenus, using *T* = dS/r and the minimum/maximum dS for each. Trees were visualized using the {ape} package (Paradis et al. 2004). Ancestral state reconstructions for genome size were completed using fastAnc from {phytools} (Revell 2012). Indels and SNPs were characterized among *Houzingenia* using the Genome Analysis ToolKit (Van der Auwera et al. 2013) and the *G. raimondii* reference sequence (Paterson et al. 2012). SNP introgression was measured by both individual SNP proportions (see https://github.com/IGBB/D_Cottons_USDA/; last accessed December 18, 2018) and ANGSD (Korneliussen et al. 2014). Indel effects on genes were measured by SnpEff (Cingolani, Platts, et al. 2012) and SnpSift (Cingolani, Patel, et al. 2012).

Gene orthology among species was determined via OrthoFinder (Emms and Kelly 2015), and copy numbers per species/gene family was derived from the resulting orthologous clusters. Copy number evolution was modeled using Count (Csurös 2010), which uses a likelihood-based phylogenetic birth-and-death model to estimate gene family sizes along edges and subsequently reconstruct ancestral states. Bootstrap-like replicates were estimated by resampling (with replacement) for 1,000 permutations.

#### Repetitive Sequence Characterization

Reads from only one of the paired-end files (i.e., R1) were filtered and trimmed via Trimmomatic version 0.33 (Bolger et al. 2014) to a uniform 85 nt (https://github.com/IGBB/D_Cottons_USDA; last accessed December 18, 2018), and then randomly subsampled to represent a 1% genome size equivalent (GSE) for each individual (Wendel et al. 2002; Hendrix and Stewart 2005). These 1% GSEs were combined as input into the RepeatExplorer pipeline (Novák et al. 2010, 2013), which has been successfully used to profile genomic repeats using low-coverage, short read sequencing. Only clusters which contain at least 0.01% of the total input sequences (i.e., 387 reads from a total input of 3,872,016 reads) were retained for annotation as per Grover 2018 (Grover et al. 2017), which uses the RepeatExplorer implementation of RepeatMasker (Smit et al.) and a custom cotton-enriched repeat library. Genome occupation of each broad repeat type was calculated (in megabases; Mb) for each genome/accession based on the 1% genome representation of the sample and the standardized read length of 85 nt.

Patterns of repeat content per genome were determined using the abundance of each cluster in a multivariate data set. Initial visualization of the data was conducted in R (R Core Team 2018) using Principle Coordinate Analysis on read counts, either log normalized (to compare overall patterns of repeats) or normalized by genome size (to compare proportional cluster size). Differential abundance in cluster occupation was iteratively calculated at increasing phylogenetic depths to understand the evolution of repeat types at different temporal scales. That is, differentially abundant clusters were determined 1) within species, 2) between sister taxa, and 3) between deeper phylogenetic nodes. For each cluster, the ancestral state was reconstructed and used for comparison in the next analysis. Ancestral state reconstructions were completed using fastAnc for reconstruction (Revell 2012) and the fitContinuous function of {Geiger} (Harmon et al. 2008) for visualization. All analyses are available at (https://github.com/IGBB/D_Cottons_USDA; last accessed December 18, 2018).

### Repeat Heterogeneity and Relative Age

Relative cluster age was approximated using the among-read divergence profile of each cluster, as previously used for *Fritillaria* (Kelly et al. 2015), dandelion (Ferreira de Carvalho et al. 2016), and *Kokia*/*Gossypioides* (Grover et al. 2017), sister outgroup genera to *Gossypium*. Briefly, cluster-by-cluster all-versus-all BLASTn (Camacho et al. 2009; Boratyn et al. 2013) searches were conducted using the same BLAST parameters implemented in RepeatExplorer. A pairwise percent identity histogram was generated for each cluster, and regression models were used to describe the trend (i.e., biased toward high-identity, “young” or lower-identity, “older” element reads) using Bayesian Information Criterion (Schwarz 1978) to select the model with the most confidence. Specific parameters can be found in Grover et al. (2017) and at https://github.com/IGBB/D_Cottons_USDA, last accessed December 18, 2018. The read similarity profile was automatically evaluated for each cluster to determine if the reads trend toward highly similar “young” or more divergent “older” reads. These profiles generally consist of six different trends: 1) positive linear regression (“young”); 2) absence of linear regression (“old”); 3) negative linear regression (“old”); 4) positive quadratic vertical parabola, trend described by right-side of vertex (“young”); 4b) positive quadratic vertical parabola, trend described by left-side of vertex (“old”); 5) negative quadratic vertical parabola, trend described by right-side of vertex (“old”); and 6) negative quadratic vertical parabola, trend described by left-side of vertex and vertex at >99% pairwise-identity (“old”). We note that “young” and “old” are relative designations and not indicative of absolute age.

## Results

### Genome Assemblies and Annotation

Approximately 22–65× raw coverage libraries were sequenced for at least one representative of each D-genome species (supplementary table 1, Supplementary Material online), resulting in an average of 169.4 M reads per accession. Quality filters further reduced the number of reads per sample to an average of 136.9 M (range: 67.2–260.2 M), representing an average of 33× coverage per sample. All accessions (except *Gossypiumthurberi* accession 2) were assembled via ABySS using multiple kmer values (see Materials and Methods section) and the assembly with the greatest E-size (Salzberg et al. 2012) was selected to represent each species. These representative assemblies were improved with the reference-based scaffolder Chromosomer (Tamazian et al. 2016) using the closely related *G. raimondii* genome (Paterson et al. 2012), producing assemblies that range in size from 585 to 775 Mbp (average 643 Mbp) and cover 67–85% of each genome (table 1). These metrics are comparable with those generated by the subgenus *Houzingenia*-derived reference genome (Paterson et al. 2012).

**Table 1:** Statistics for the Best Assembled Accession

Scaffold statistics	Subsection	Species	Accession	# Contigs (>= 1 kb)	Largest Contig (Mb)	Contigs >= 1 kb	Contigs >= 25 kb	Contigs >= 50 kb	Total Length	Genome Size (GS)	% Genome Covered	N50	% *N*	# Gene Models	# BUSCOs	% Recovered	# Partial BUSCO
*Austroamericana*	*G. raimondii*	Paterson et al. (2012)	1,033	70.71	761.41	754.80	753.82	761.41	880	86.5%	62.18	2%				
*Austroamericana*	*G. raimondii*	D5-8	1,431	53.83	589.99	585.87	585.79	592.04	880	67.3%	48.45	14%	30,475	1,339	93%	27
*Caducibracteata*	*G. armourianum*	D2-1-6	13,359	55.08	645.03	600.41	599.97	671.70	856	78.5%	47.59	15%	28,845	1,124	78%	55
*Caducibracteata*	*G. harknessii*	JFW	20,602	52.42	615.99	541.69	540.67	643.05	910	70.7%	43.64	7%	36,068	1,294	90%	51
*Caducibracteata*	*G. turneri*	D10-7	18,841	49.15	742.21	654.97	601.54	774.62	910	85.1%	33.33	2%	45,244	1,366	95%	17
*Erioxylum*	*G. aridum*	DRD-185	21,813	52.48	619.71	552.02	551.45	648.51	919	70.6%	42.90	8%	35,142	1,285	89%	47
*Erioxylum*	*G. lobatum*	D7-157	22,383	53.83	625.55	555.17	554.44	654.85	934	70.1%	43.64	8%	35,572	1,310	91%	41
*Erioxylum*	*G. laxum*	D9-4	16,668	60.54	689.25	623.49	621.55	720.31	934	77.1%	48.55	13%	32,375	1,321	92%	37
*Erioxylum*	*G. schwendimanii*	D11-1	18,906	52.32	623.31	526.16	513.54	651.41	929	70.1%	40.29	5%	38,314	1,348	94%	25
*Houzingenia*	*G. thurberi*	D1-35	15,309	47.60	582.19	505.27	498.80	605.21	841	72.0%	37.86	4%	37,553	1,342	93%	26
*Houzingenia*	*G. trilobum*	D8-8	14,099	44.53	562.98	483.41	474.98	586.05	851	68.9%	36.15	5%	36,663	1,321	92%	43
*Integrifolia*	*G. davidsonii*	D3D-27	16,779	48.06	603.64	517.19	506.66	629.89	910	69.2%	38.65	3%	38,755	1,237	86%	41
*Integrifolia*	*G. klotzschianum*	D3K-57	16,881	46.26	569.35	495.76	492.77	596.12	880	67.7%	37.84	5%	37,444	1,332	93%	37
*Selera*	*G. gossypioides*	D6-5	23,734	42.78	554.55	446.85	441.62	585.41	841	69.6%	33.53	4%	26,492	1,133	79%	41

Assemblies from all accessions were annotated, resulting in between 20,522 and 45,244 gene models per accession (min = 26,492 for improved assemblies), similar to the number of primary transcripts published for *G. raimondii* (Paterson et al. 2012)*.* BUSCO (Simão et al. 2015) analysis recovered over 80% of BUSCOs from nearly 80% of the improved assemblies, where a gene was considered present if more than 67% of the gene was recovered from that accession. This suggests a general completeness of the gene space, with an average of 87% complete BUSCOs recovered from each accession and less than 3.5% redundancy on average (table 1).

Chloroplast reads were also recovered from the raw data, representing an average of 3% (range: 1.46–7.27%) of the filtered sequencing reads. These were used in reference-guided assemblies against the published *Gossypiumhirsutum* chloroplast genome (Lee et al. 2006). The chloroplast genome alignment (excluding positions with ambiguity in any sequence) size was 158,996 bp, comparable with previously published cotton chloroplast genomes (Cronn et al. 2002; Chen et al. 2016). Chloroplast sequences were retained for phylogenetic analyses, and are available under Genbank accessions MH477706 through MH477724.

### Phylogenetic Relationships among New World Cottons

**Fig. 2.**
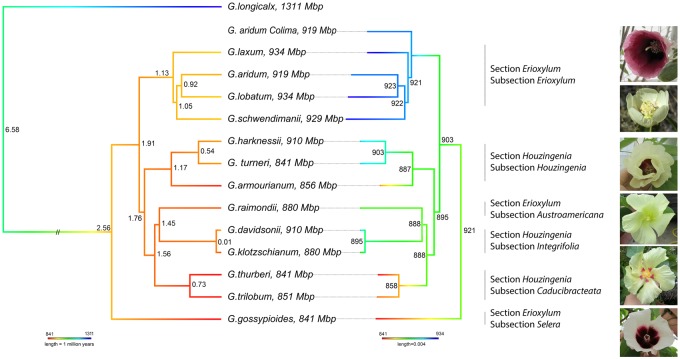
—Nuclear phylogeny of *Houzingenia* without (left) and including (right) the introgressed accession of *G. aridum* from the Mexican state of Colima. Divergence times are visualized on an ultrametric tree (left) whose colors correspond to the relative growth (blue) or reduction (red) of genome size in *Houzingenia*, as compared with the outgroup *G. longicalyx* (*Longiloba*). Inferred ancestral genome sizes are displayed on a proportional tree (right) whose colors correspond to the degree of change within *Houzingenia* alone. Phylogenetic methods, divergence time estimates, and ancestral state reconstruction details are in the methods.

Phylogenetic relationships among *Houzingenia* species were revisited using a concatenation of 7,595 dispersed nuclear genes containing a minimum of one accession per species (see filtering criteria in methods). After removing any alignment position with >10% ambiguity, >20.3 million nucleotides derived from all 13 chromosomes remained for 22 *Houzingenia* accessions and for the outgroup, *G. longicalyx* (subgenus *Longiloba*). Maximum likelihood reconstruction of the phylogenetic relationships among species largely recovers established section and subsection relationships (fig. 2). As previously reported, whereas both sections of the subgenus, that is, *Houzingenia* and *Erioxylum*, exhibit polyphyly, the individual subsections are either monophyletic or monotypic (fig. 2). Species relationships are largely congruent with the most recent phylogenetic inferences for the subgenus using nuclear genes (Álvarez et al. 2005), both of which differ from the subgenus SSR dendrogram (Ulloa 2014) in the placement of several taxa, including *G. raimondii*, *G. davidsonii*, and *G. gossypioides*.

**Fig. 3.**
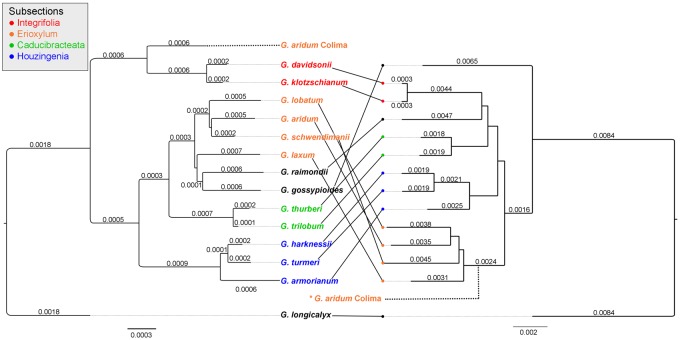
—Comparison of phylogeny from reference-guided assembly of chloroplast-derived reads in *Houzingenia* (left; ML-derived branch lengths are listed) and the nuclear phylogeny (right). The position of *G. aridum* Colima on the nuclear phylogeny (right) has been added to the figure with a dotted line, as presence of this accession “attracts” *G. schwendimanii* to its position thereby distorting the topology; the nonintrogressed topology is pictured here. The chloroplast phylogeny shown here was derived from WGS-derived whole chloroplast sequences (see Materials and Methods section); this tree topology was also recovered from a concatenated chloroplast gene-only phylogenetic analysis that includes all published sequences in Genbank (see https://github.com/IGBB/D_Cottons_USDA, last accessed December 18, 2018 for details). Each node in the chloroplast phylogeny had 100% bootstrap support. Whereas within subsection associations among species are supported between the trees (in colors), the relationship among subsections varies between the two molecule types.

Notably, one of the two *G. aridum* accessions included (D4-12C from Colima, Mexico; PI 530897) is placed sister to the rest of the arborescent cottons of subsection *Erioxylum* and not sister to the *G. aridum* accession from Jalisco (Álvarez et al. 2005). This observation recapitulates that of Alvarez and Wendel (2006), which used AFLPs to evaluate 143 individuals from 50 populations of subsection *Erioxylum* species and the related subsection, *Integrifolia*, which was previously identified as a source of cytoplasmic introgression in Colima *G. aridum* accessions (Dejoode and Wendel 1992). Indeed, phylogenetic analysis of the entire chloroplast for *Houzingenia* species (fig. 3) concurs with previous chloroplast restriction site analysis (Wendel and Albert 1992), which suggest that the Colima *G. aridum* accession (D4-12C) has an *Integrifolia* derived cytoplasm. It is interesting to note that diversity analyses of subsection *Erioxylum* using SSR markers (Ulloa et al. 2006; Feng et al. 2011; Ulloa 2014) suggest that the circumscription of *G. aridum* may include previously undescribed species, a potential alternative hypothesis to introgression. SNP analyses of the two *G. aridum* accessions included here suggest that the Colima accession does retain evidence of nuclear introgression. This was determined using an ABBA–BABA test (Sousa and Hey 2013; Korneliussen et al. 2014) with both accessions of *G. aridum* (H1 and H2), *G. davidsonii* as the source of introgression (H3), and *G. gossypioides* as the ancestral state (outgroup). This analysis confirms ancient admixture resulting in introgression from a *G. davidsonii*-like species into *G. aridum* Colima (*Z* = −3.64, representing significant deviation from the mean).

To further characterize the extent of nuclear introgression in *G. aridum* Colima, we compared the number of inferred introgressed SNPs (i.e., derived SNPs shared between *G. aridum* Colima and *G. davidsonii*) against the number of SNPs where *G. aridum* Jalisco (nonintrogressed) shares a derived state with *G. davidsonii*. This tabulation (table 2) gives the same results as the ABBA–BABA test (χ^2^*P*-value = 0), confirming nuclear introgression from subsection *Integrifolia* into *G. aridum* from Colima. When the data are partitioned by chromosome, about half of the chromosomes show an excess of derived SNPs compared with their counterpart in the nonintrogressed *G. aridum* from Jalisco (table 2), indicating that perhaps the genomic distribution of surviving introgressed regions has been uneven. Although the number of genes showing derived SNPs, and hence a residue of introgression, is not significantly different between the two *G. aridum* accessions, the Colima *G. aridum* does exhibit an excess of SNPs *in genes* (*P* = 0.0015). The latter is important in that these SNPs, while limited, both have high confidence in their orthology and support the broader conclusion that ancient nuclear introgression occurred in the Colima populations of *G. aridum*.

**Table 2:** Number of Shared, Derived Nuclear SNPs between *G. davidsonii* (Integrifolia) and *G. aridum* Accessions from Colima and Jalisco

	*G. aridum* (Colima)	*G. aridum* (Jalisco)	*P* Value
Overall SNPs	188,472	182,563	0.0005
Chr01 SNPs	12,808	12,808	1.0000
Chr02 SNPs	17,118	17,094	0.8941
Chr03 SNPs	11,956	11,353	0.0005
Chr04 SNPs	17,292	16,643	0.0005
Chr05 SNPs	18,950	18,065	0.0005
Chr06 SNPs	11,013	10,732	0.0600
Chr07 SNPs	15,822	14,649	0.0005
Chr08 SNPs	12,904	12,911	0.9795
Chr09 SNPs	15,131	14,922	0.2399
Chr10 SNPs	17,585	16,895	0.0005
Chr11 SNPs	15,741	14,941	0.0005
Chr12 SNPs	8,600	8,636	0.8081
Chr13 SNPs	13,552	12,914	0.0005
Genic SNPs	7,843	7,419	0.0015
Number of genes	4,808	4,721	0.3733

Note.—Previous research indicates that Colima *G. aridum* has Integrifolia-derived cytoplasm and nuclear sequences. *Gossypium gossypioides* was used for ancestral states.

In addition to the evidence for introgression into Colima *G. aridum*, comparison between the nuclear and chloroplast phylogenies supports previous observations of *Austroamericana*-derived introgression in subsection Selera (G. gossypioides) *Gossypium gossypioides* is unusual within *Houzingenia* as it has likely undergone two separate instances of introgression: 1) the more recent chloroplast introgression noted here and elsewhere (Wendel and Albert 1992; Cronn et al. 2003; Cronn and Wendel 2003), and 2) nuclear introgression, as evidenced by the presence of African cotton-like ITS (Wendel et al. 1995) and repetitive DNA (Zhao et al. 1998). Clear evidence of chloroplast-nuclear conflict is seen in the analyses here, congruent with previous observations, which is resolved when the putatively introgressed accessions are removed (data not shown). Evidence for nuclear introgression is less clear (see below) and warrants additional analyses involving more *Gossypium* species, which is beyond the scope of the present paper.

### Recent Divergence in Subgenus *Houzingenia* Is Reflected in the Low Rate of Molecular Evolution

Divergence times were estimated for the thirteen extant *Houzingenia* species (fig. 2) using the synonymous substitution rate for the Malvaceae, as described in Grover et al. (2017). Subgenus *Houzingenia* diverged an estimated 6.58 Ma from the remaining cotton subgenera (represented by *Longiloba*), a value within prior estimates (Senchina et al. 2003). The lineage leading to *G. gossypioides* was inferred as the first to diverge from the rest of the subgenus, approximately 2.56 Ma (fig. 2), although we note that there may be additional error in this estimation arising from cryptic nuclear introgression in *G. gossypioides*. For this reason, the time estimates for all nodes (including *G. gossypioides*) were calibrated using the next most basal node, which separates section *Erioxylum* subsection *Erioxylum* from the remaining subgenus (see Materials and Methods section), in conjunction with the root. Most species are inferred to have diverged relatively recently, within the last 0.5–2 Myr, with the notable exception of *G. davidsonii* and *G. klotzschianum*, here estimated to share an ancestor that is an order of magnitude more recent than previously suggested by allozyme and chloroplast restriction site analysis (Wendel and Percival 1990). Their near-identical nature is reflected in both their estimated nuclear branch lengths (0.0003 substitutions per site vs 0.0018–0.0065 on other terminal branches) and their rates of substitution (0.0000–0.0048 dS and 0.0000 dN; table 3). While this close relationship between *G. davidsonii* and *G. klotzschianum* has been reported previously (Wendel and Percival 1990), this is the first modern estimate of genome-wide divergence between these two species.

**Table 3:** Median Synonymous (Bottom) and Nonsynonymous (Top) Mutation Rates between *Houzingenia* Species

		*Austroamericana*	*Caducibracteata*	*Caducibracteata*	*Caducibracteata*	*Erioxylum*	*Erioxylum*	*Erioxylum*	*Erioxylum*	*Houzingenia*	*Houzingenia*	*Integrifolia*	*Integrifolia*	*Selera*
		*G. raimondii*	*G. armourianum*	*G. harknessii*	*G. turneri*	*G. aridum*	*G. laxum*	*G. lobatum*	*G. schwendemanii*	*G. thurberi*	*G. trilobum*	*G. davidsonii*	*G. klotzschianum*	*G. gossypioides*
*Austroamericana*	*G. raimondii*		0.0194 (0.0115–0.0334)	0.0215 (0.0132–0.0375)	0.0216 (0.0133–0.0361)	0.0238 (0.0149–0.0403)	0.0208 (0.0130–0.0343)	0.0241 (0.0150–0.0401)	0.0228 (0.0149–0.0359)	0.0166 (0.0099–0.0285)	0.0177 (0.0101–0.0314)	0.0178 (0.0106–0.0305)	0.0173 (0.0102–0.0295)	0.0253 (0.0170–0.0394)
*Caducibracteata*	*G. armourianum*	0.0023 (0.0008–0.0041)		0.0144 (0.0072–0.0301)	0.0140 (0.0069–0.0286)	0.0229 (0.0134–0.0402)	0.0198 (0.0117–0.0339)	0.0229 (0.0137–0.0398)	0.0216 (0.0133–0.0349)	0.0166 (0.0095–0.0294)	0.0178 (0.0099–0.0324)	0.0194 (0.0115–0.0338)	0.0187 (0.0111–0.0323)	0.0241 (0.0155–0.0394)
*Caducibracteata*	*G. harknessii*	0.0025 (0.0011–0.0046)	0.0016 (0.0000–0.0033)		0.0080 (0.0000–0.0235)	0.0245 (0.0149–0.0419)	0.0225 (0.0138–0.0383)	0.0246 (0.0149–0.0410)	0.0241 (0.0149–0.0403)	0.0192 (0.0112–0.0348)	0.0201 (0.0118–0.0367)	0.0218 (0.0132–0.0381)	0.0211 (0.0129–0.0364)	0.0266 (0.0175–0.0438)
*Caducibracteata*	*G. turneri*	0.00260 (0.0012–0.0047)	0.0016 (0.0000–0.0033)	0.0001 (0.0000–0.0025)		0.0248 (0.0154–0.0426)	0.0214 (0.0134–0.0362)	0.0248 (0.0153–0.0415)	0.0227 (0.0148–0.0356)	0.0186 (0.0113–0.0313)	0.0186 (0.0111–0.0315)	0.0201 (0.0126–0.0328)	0.0200 (0.0124–0.0327)	0.0256 (0.0170–0.0393)
*Erioxylum*	*G. aridum*	0.0027 (0.0012–0.0049)	0.0026 (0.0011–0.0047)	0.0028 (0.0013–0.0051)	0.0029 (0.0013–0.0053)		0.0150 (0.0076–0.0296)	0.0154 (0.0079–0.0297)	0.0166 (0.0090–0.0314)	0.0216 (0.0132–0.0372)	0.0235 (0.0138–0.0411)	0.0236 (0.0149–0.0398)	0.0235 (0.0149–0.0397)	0.0287 (0.0187–0.0460)
*Erioxylum*	*G. laxum*	0.0026 (0.0011–0.0045)	0.0023 (0.0009–0.0043)	0.0026 (0.0012–0.0047)	0.0027 (0.0012–0.0049)	0.0015 (0.0000–0.0033)		0.0146 (0.0076–0.0286)	0.0137 (0.0076–0.0236)	0.0183 (0.0112–0.0302)	0.0192 (0.0118–0.0321)	0.0204 (0.0127–0.0333)	0.0199 (0.0125–0.0322)	0.0247 (0.0164–0.0382)
*Erioxylum*	*G. lobatum*	0.0028 (0.0013–0.0050)	0.00260 (0.00120–0.00480)	0.0028 (0.0013–0.0051)	0.0030 (0.0014–0.0053)	0.0016 (0.0000–0.0034)	0.0016 (0.0000–0.0033)		0.0166 (0.0094–0.0304)	0.0215 (0.0132–0.0366)	0.0231 (0.0139–0.0400)	0.0238 (0.0148–0.0401)	0.0235 (0.0146–0.0391)	0.0284 (0.0186–0.0438)
*Erioxylum*	*G. schwendemanii*	0.0028 (0.0013–0.0049)	0.0026 (0.0012–0.0046)	0.0030 (0.0014–0.0052)	0.0028 (0.0014–0.0050)	0.0018 (0.0000–0.0037)	0.0017 (0.0000–0.0032)	0.0019 (0.0001–0.0037)		0.0202 (0.0127–0.0313)	0.0206 (0.0132–0.0331)	0.0219 (0.0146–0.0339)	0.0218 (0.0142–0.0335)	0.0264 (0.0180–0.0391)
*Houzingenia*	*G. thurberi*	0.0020 (0.0006–0.0037)	0.0020 (0.0006–0.0039)	0.0023 (0.0009–0.0043)	0.0023 (0.0010–0.0042)	0.0025 (0.0011–0.0047)	0.0023 (0.0010–0.0041)	0.0026 (0.0012–0.0046)	0.0025 (0.0012–0.0044)		0.0064 (0.0023–0.0144)	0.0153 (0.0091–0.0259)	0.0151 (0.0089–0.0252)	0.0229 (0.0152–0.0347)
*Houzingenia*	*G. trilobum*	0.0021 (0.0007–0.0040)	0.0020 (0.0007–0.0040)	0.0024 (0.0010–0.0045)	0.0024 (0.0010–0.0045)	0.0027 (0.0012–0.0050)	0.0024 (0.0010–0.0044)	0.0027 (0.0012–0.0050)	0.0026 (0.0012–0.0046)	0.0007 (0.0000–0.0019)		0.0162 (0.0094–0.0281)	0.0159 (0.0089–0.0272)	0.0236 (0.0153–0.0367)
*Integrifolia*	*G. davidsonii*	0.0021 (0.0007–0.0040)	0.0022 (0.0009–0.0042)	0.0025 (0.0011–0.0045)	0.0026 (0.0012–0.0046)	0.0027 (0.0012–0.0050)	0.0025 (0.0011–0.0045)	0.0028 (0.0013–0.0050)	0.0027 (0.0013–0.0048)	0.0019 (0.0007–0.0036)	0.0020 (0.0007–0.0038)		0.0000 (0.0000–0.0048)	0.0251 (0.0165–0.0377)
*Integrifolia*	*G. klotzschianum*	0.0020 (0.0006–0.0038)	0.0021 (0.0008–0.0041)	0.0025 (0.0010–0.0045)	0.0024 (0.0011–0.0045)	0.0027 (0.0012–0.0049)	0.0024 (0.0011–0.0044)	0.0028 (0.0013–0.0049)	0.0027 (0.0013–0.0046)	0.0018 (0.0005–0.0034)	0.0019 (0.0006–0.0036)	0.0000 (0.0000–0.0000)		0.0246 (0.0162–0.0368)
*Selera*	*G. gossypioides*	0.0030 (0.0014–0.0051)	0.0028 (0.0013–0.0050)	0.0032 (0.0015–0.0055)	0.0032 (0.0016–0.0055)	0.0032 (0.0016–0.0057)	0.0030 (0.0015–0.0052)	0.0033 (0.0016–0.0057)	0.0032 (0.0017–0.0054)	0.0028 (0.0013–0.0048)	0.0028 (0.0014–0.0050)	0.0030 (0.0015–0.0051)	0.0029 (0.0014–0.0050)	

Note.—Values in parentheses represent upper- and lower quartile, respectively.

Genome-wide rates of molecular evolution among *Houzingenia* species were calculated for all species comparisons (table 3). As expected, pairwise synonymous mutation rates (dS, average = 0.0213 substitutions/site) were approximately an order of magnitude greater than the nonsynonymous mutation rates (dN, average = 0.0026; table 3). Synonymous mutation rates varied from 0.0000 between the two extant members of subsection *Integrifolia*, *G. davidsonii* and *G. klotzschianum*, to 0.0287 between *G. aridum* and the earliest-diverging member of *Houzingenia*, *G. gossypioides*. When considering divergence time between species, the dS range narrows to between 0 and 0.017 substitutions/site/million years with 94% of the comparisons falling between dS/Myr = 0.009–0.013. A single dS comparison, *G. davidsonii* and *G. klotzschianum*, was less than this range. No pattern was evident in the four values that exceeded this range. Similarly, dN varied from 0.000 between *G. davidsonii* and *G. klotzschianum* to 0.0033 between *Gossypiumlobatum* and *G. gossypioides*, again reflecting the ancient divergence of *G. gossypioides* with the rest of *Houzingenia*. When standardized by time, the range narrows to dN = 0–0.0018, with 90% between dN = 0.0011–0.0015. Again, the *Integrifolia* species occupied the lowest dN value; however, notably, the dN value for *Gossypiumturneri* versus *G. harknessii* was similarly small (dN = 0.0002). This stands in contrast to the dS value for the pair, which was comparably large at dS = 0.0148 (table 3).

### Transposable Elements in *Houzingenia* Are Older and Concordant With Small Genome Sizes

**Fig. 4.**
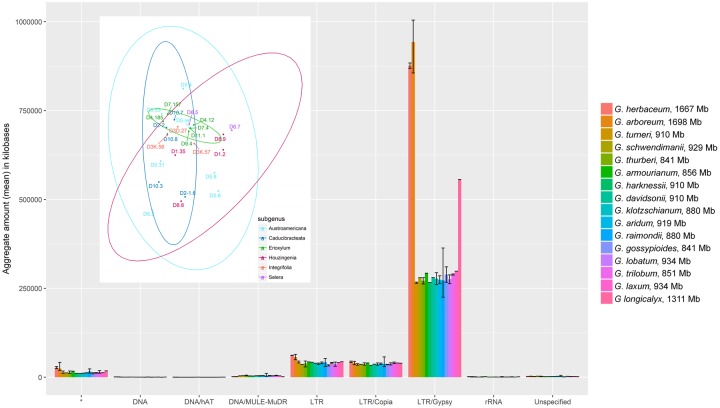
—Mean transposable element content for each category in each species of *Houzingenia*, as well as representatives from *Gossypium* and *Longiloba*. The (average) aggregate number of kilobases represented by each transposable element category for each species (genome sizes included next to species names). Transposable elements were broadly categorized into categories and their representation per species summarized, with the minimum and maximum per species included. Repetitive clusters that did not match any sequence in the database are denoted by the asterisk, whereas repetitive clusters that matched conflicting categories were classified as “Unspecified.” **Inset**: Multidimensional comparison of *Houzingenia* species based on repetitive content. Species are designated by their numbered designations: D1 (*G. thurberi*), D2-1 (*G. armourianum*), D2-2 (*G. harknessii*), D3D (*G. davidsonii*), D3K (*G. klotzschianum*), D4 (*G. aridum*), D5 (*G. raimondii*), D6 (*G. gossypioides*), D7 (*G. lobatum*), D8 (*G. trilobum*), D9 (*G. laxum*), D10 (*G. turneri*), and D11 (*G. schwendimanii*). Confidence intervals (95%) are shown for subgenera with a minimum of four representatives.

Similar to previous reports (Paterson et al. 2012), repetitive DNAs contribute roughly half of the total genome sequence for all species in subgenus *Houzingenia*, from an average of 39.4% in *G. harknessii* to 46.9% in *Gossypiumarmourianum*. Like most flowering plants, a vast majority of this sequence is due to the prevalence of Class II *gypsy* elements, which comprise 29.2–34.3% of the total genome size for any *Houzingenia* species (fig. 4). Multidimensional TE profile visualization using both log-transformed and percent-genome size standardized counts showed considerable overlap among species, and even among subsections (fig. 4). Multivariate t-distribution confidence ellipses (as implemented in ggplot2) are drawn for each subsection, all of which overlap with at least one other subsection. Even those subsections where sampling was insufficient to generate of a confidence ellipse (i.e., *Selera* and *Integrifolia*), the plotted data points are contained within the occupied space of another subsection (fig. 4, inset). *Selera*, for example, is contained within the confidence ellipse for both all other subsections, as is *Integrifolia*. Likewise, few repetitive elements (14 elements at *P* < 0.5, 13 *gypsy* and 1 undefined) differ significantly in copy number among *Houzingenia* species. This apparent overlap in repetitive element profiles is also suggested by the relative amounts of each transposable element category among subsections (fig. 4).

To compare the overlap among subsections, we performed a Procrustes ANOVA, as implemented in the R package {geomorph} (Adams and Otárola-Castillo 2013). For this analysis, we compared each subsection using all representatives of that subsection as indicators of variance. Few comparisons showed statistically significant differences, with the patterns of repetitive abundance differing only between *Austroamericana* and *Caducibracteata* and between *Integrifolia* and *Selera* (*P* < 0.05).

The absolute amount of sequence attributable to each type of TE category is similar among *Houzingenia* species and is distinguishable from the African subgenera, primarily for *gypsy* elements (fig. 4). The total amount of *gypsy* elements predicted for the African species is far greater (average 878 Mb vs 277 MB, respectively), which is expected given previous analyses of cotton transposable elements (Hawkins et al. 2006, 2009; Grover et al. 2007). The total amount of predicted MULE/MuDR-like elements, however, is greater for *Houzingenia* (average 4.4 Mb vs 1.6 Mb in the African subgenera) even despite the large difference in genome size, an observation not previously reported. These patterns persist even when comparing TEs as a function of genome size (supplementary fig. 1, Supplementary Material online), with two additional observations. First, the large error bars for *gypsy* amount in *G. raimondii* become more pronounced. Inspection of the total amounts for this species suggests that there is a single accession (*G. raimondii* accession D5-6) that has remarkably more *gypsy* elements than the remaining conspecifics. Whereas approximately 30% of *gypsy* clusters in *G. raimondii* accession 6 are found in excess (relative to the other accessions), less than quarter of these contribute >1 Mb additional sequence, indicating minor to modest relative proliferation in most cases. Interestingly, however, a single *gypsy* cluster (cluster 78) comprises 4.8 Mb additional sequence in *G. raimondii* accession 6 relative to the conspecific with the closest amount (12.6 Mb in *G. raimondii* accession 6 vs 7.8 Mb in accession 8). The average for this cluster, including *G. raimondii* accession 6, is only 5.2 Mb. These observations suggest that the *gypsy* element represented by cluster 78 has been recently active in the *G. raimondii* genome, achieving significant success in at least one lineage.

Previous research on *G. raimondii* (subsection *Austroamericana*) demonstrated a relative lack of lineage-specific amplification with concomitant removal of a prolific cotton *gypsy* element as a mechanism for genome downsizing in *G. raimondii* (Hawkins et al. 2009). Congruent with these results, most of the clusters recovered here are composed primarily of “older” reads (68.6–78.6% per accessions), that is, reads more divergent than expected for recently active transposable elements. Ancestral state reconstruction of individual clusters, however, demonstrates both amplification and removal concomitant with the inferred changes in overall genome size (fig. 2; supplementary fig. 2, Supplementary Material online). Most clusters are “older,” with 39% of clusters comprised solely of “older” repeats and the remaining clusters most frequently showing recent amplification in one to few lineages (supplementary fig. 3, Supplementary Material online).

### Genome Differentiation via Insertions and Deletions

Small-scale insertions and deletions are a common form of sequence variation, with the potential to alter regulatory as well as coding regions (Britten et al. 2003; Halligan et al. 2013; Tuğrul et al. 2015; Lin et al. 2017). While this is particularly true for large-scale, TE-associated indels (e.g., transposable element insertions), the formation of smaller indels can also vary among related species (Sato et al. 2012; Chintalapati et al. 2017; Kapusta et al. 2017). Accordingly, we evaluated the extent of indel evolution among *Houzingenia* species, using the *G. raimondii* genome as the reference state and polarized using *G. longicalyx* (subgenus *Longiloba*). Phylogenetic analysis of coded indels as multistate characters (see Materials and Methods section) reproduces the nuclear phylogeny, suggesting that indel formation largely corresponds to species relationships. In total, small indels were present at 1,149,943 positions in at least one of the 13 *Houzingenia* species (relative to the outgroup *Longiloba*). Within *Houzingenia*, indels distinguish one or more species at 761,746 locations. The range in number of these distinguishing indels per chromosome varies by over 31,000 events, from 40,747 indels on chromosome 12 to 72,303 indels on chromosome 9, the smallest and longest chromosomes, respectively. Relative to the length of each chromosome, the gap narrows to between 779 indels/Mb on chromosome 5 and 1,174 indels/Mb on chromosome 8, a difference of 395 indels/Mb. Indels ranged in size from 1 to 270 nt, with an average of 6.2 nt/indel. Whereas the size of the largest indel detected varied among chromosomes, the average indel size per chromosome ranged narrowly from 5.7 to 6.7 nt/indel (table 5).

**Table 5:** Indels in *Houzingenia* Relative to the Outgroup *G. longicalyx* (*Longiloba*), Partitioned by Chromosome and by Species

	# Indels	Chromosome Length (Mb)	# Indels/Mb	Average Indel Size (nt)	Maximum Indel Size (nt)
Chromosome 1	63,848	55.9	1,143	6.3	187
Chromosome 2	60,823	62.8	969	5.9	230
Chromosome 3	48,607	45.8	1,062	6.1	262
Chromosome 4	58,550	62.2	942	6.0	182
Chromosome 5	49,943	64.1	779	5.7	164
Chromosome 6	58,156	51.1	1,139	6.5	173
Chromosome 7	67,740	61.0	1,111	6.4	270
Chromosome 8	67,069	57.1	1,174	6.5	188
Chromosome 9	72,303	70.7	1,022	6.7	183
Chromosome 10	59,521	62.2	957	6.0	214
Chromosome 11	62,707	62.7	1,000	6.1	220
Chromosome 12	40,747	35.4	1,150	6.0	181
Chromosome 13	51,732	58.3	887	6.0	197
		# SNPs	# Indels	SNPs:Indels	
*G. raimondii*	D5-8	7,909,366	451,713	18	
*G. armourianum*	D2-1-6	7,525,371	442,985	17	
*G. harknessii*	D2-2	8,140,633	474,421	17	
*G. turneri*	D10-7	8,155,064	475,161	17	
*G. aridum*	D4-185	8,555,662	487,561	18	
*G. lobatum*	D7-157	8,651,866	490,322	18	
*G. laxum*	D9-4	8,015,127	462,728	17	
*G. schwendimanii*	D11-1	8,606,096	491,961	17	
*G. thurberi*	D1-35	8,139,420	478,238	17	
*G. trilobum*	D8-8	8,232,774	482,728	17	
*G. davidsonii*	D3D-27	8,539,202	493,939	17	
*G. klotzschianum*	D3K-57	8,545,127	494,072	17	
*G. gossypioides*	D6-5	8,359,287	513,538	16	

Among accessions and chromosomes, the number of indels/Mb is relatively similar (98–260 indels/Mb on *G. raimondii* chromosome 1 and *G. gossypioides* chromosome 6, respectively; supplementary table 2, Supplementary Material online), but statistically distinct (χ^2^*P* < 0.01). Deletions generally outweigh insertions for each chromosome/accession combination, both with respect to number (2-fold) and length (2.5- to 5-fold; supplementary table 2, Supplementary Material online). This results in a net loss of between 278 and 555 kb per accession (*G. raimondii* and *Gossypiumtrilobum*, respectively; average = 439 kb). Compared with the rate of nucleotide substitution, the rate of indel events is much lower and is approximately equivalent among species (from 16 to 18 nucleotide changes per indel event; supplementary table 2, Supplementary Material online). The rate of indel formation among chromosomes and accessions varies slightly more than the overall rate, from 14 to 23 substitutions per indel. Whereas no obvious patterns exist in this respect, the earliest-diverging lineage, *G. gossypioides*, consistently has more indels relative to SNPs, possibly as a consequence of its introgressed history (Wendel et al. 1995; Cronn et al. 2003). Whereas our understanding of the pattern and rate of indel formation among species would be increased through whole genome alignment of higher quality, de novo genome sequences rather than the resequenced genomes utilized here, our preliminary data suggest that differences in small indel evolution may not have a significant effect at this scale; however, these results do support the idea that small deletions may be able to partially counteract genome size growth by TE amplification and small insertions.

The genic consequences for these indels were evaluated for the 37,223 gene models in the *G. raimondii* reference (Paterson et al. 2012). Less than 1.5% of indels (15,786) had any in-gene effects in any species, of which 12,679 (19%) only result in a single amino acid gain or loss (1,333 and 1,663 indels, respectively). Nearly 50% of exonic indels resulted in a frameshift mutation, 8% of which had additional consequences (e.g., gain or loss of start, stop, or splice signal). Over 27% affected the protein length only, with a slight bias (2:1) toward inframe deletions and only 1.4% of these affecting the start or stop codons.

In total, 9,342 genes were affected by indels in at least one species; however, most species exhibited indel-induced genic changes in an average of 2,700 genes, of which approximately 600 induce length changes only. Notably, whereas the *G. raimondii* accession sequenced had the fewest indels detected in genes, 1.8% of the gene models were nevertheless affected in this accession. Given the relative uniformity of *G. raimondii* in protein-coding sequences (Wendel JF, unpublished data), this may represent the amount of error inherent in the indel analysis due to the bioinformatic identification of indels or to the gene models represented in the published genome.

### Genome Differentiation via Copy Number Evolution

Recently, the extent of variation in gene content within and among plant species has been conceptualized in terms of the “pan-genome,” which refers to the suite of genes present within or among closely related species (Lai et al. 2010; Hirsch et al. 2014; Li, Zhou, et al. 2014; Lin et al. 2014; Schatz et al. 2014; Golicz, Batley, et al. 2016; Golicz, Bayer, et al. 2016; Pinosio et al. 2016; Montenegro et al. 2017). Here, we begin to evaluate the scope of a *Houzingenia*-specific pan-genome by modeling genic copy number evolution. Homologous gene clusters generated via OrthoFinder were used as input in Count (Csurös 2010), which has been developed to conduct evolutionary analyses of homologous family sizes in a phylogenetic context, including inferring the rate of gene gain and loss for each phylogenetic branch. We found that the inferred rate of loss for a given lineage was consistently greater than the rate of gain (with the exception of *G. turneri*). Among lineage rate variability was observed for both inferred losses and gains; however, the magnitude of variability in the inferred rate of losses was far greater (0.05–0.41 losses per branch) than in gains (0.00–0.13 gains/branch). Standardizing these rates to account for variability in nucleotide substitution rates (as a proxy for time) reduces the difference in variability between the rate of loss (0.06–0.31) and gain (0.00–0.25).

**Fig. 5.**
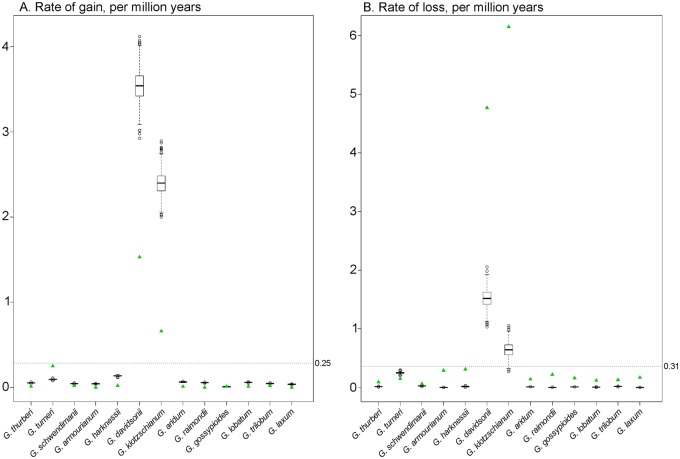
—Rate of gene gain or loss, per million years. Boxplot distributions show distribution of gene gain (*A*) or loss (*B*), per species, as inferred from the resampled data (see Materials and Methods section). Inferred rates of gain or loss from the total data set are displayed as green triangles. Inferred rates for both gain and loss are substantially higher in *G. davidsonii* and *G. klotzschianum*, likely due to rate inflation based on the substantially shorter branches leading to these taxa.

Because these summarized rates of loss and gain could be influenced by the effects of a few orthogroups, we performed a random resampling of the data and plotted the distribution for losses and gains relative to the observed rate (fig. 5). Generally, with the exception of *G. turneri*, the inferred rate of loss greatly exceeded the resampled range, indicating the presence of highly influential orthogroups. The inverse, however, was observed in the resampled gain data, where the inferred rates typically were less than the resampled range. These results suggest that the rate of gene loss and gain in these lineages may be sensitive to changes in family size for a few orthogroups. A caveat, however, is that these inferences are based on orthogroup membership, which are clusters of closely related genes (i.e., gene families). In most cases, these orthogroups will have few members; however, in some cases, orthogroup membership will rise to many members in some species, such that there is an order of magnitude difference between species for those clusters. Therefore, while these results indicate patterns that may exist in copy number evolution among closely related species, further analyses involving synteny to determine strict orthology are required to fully understand the nuances of copy number evolution across time and among lineages.

## Discussion

The New World diploid cottons comprise a monophyletic assemblage of primarily Mexican, D-genome species that are of interest because of their involvement in origin of the allopolyploid (AD-genome) cottons, which include the commercially important species *G. hirsutum* (upland cotton) and *Gossypiumbarbadense* (Pima cotton) (reviewed in Wendel and Grover, 2015). In addition, previous work has indicated that several species in the group have complex evolutionary histories involving cryptic interspecific hybridization and introgression (all earlier citations). Here, we employed whole genome resequencing representing all species in the subgenus to characterize the evolutionary history of the subgenus and provide insight into the molecular evolution among closely related species.

### Phylogenetic and Geographic History of Subgenus *Houzingenia*

Earlier investigations of phylogenetic relationships within the subgenus were based on relatively shallow genomic surveys (e.g., several nuclear genes, or cpDNA restriction site analysis) or incomplete taxonomic sampling. Here, we used 7,595 nuclear genes from throughout the genome as well as whole-chloroplast genome sequences to re-evaluate phylogenetic relationships. Our analyses generally support previously established sectional and subsection relationships (fig. 2), and that neither taxonomic section, that is, *Houzingenia* and *Erioxylum*, is monophyletic. Among the most notable inferences enabled by the phylogenetic reconstruction based on the 7,595 nuclear genes are the following: 1) *G.gossypioides* arose from the earliest-diverging lineage within the clade, as suggested by earlier work using rather limited genomic sampling. This is a rare, highly localized species from Oaxaca, Mexico, with an unusual genomic composition that appears to reflect accumulated reticulations with other species (this topic addressed below). 2) The Mexican complex of arborescent species (to 10 or more meters in height) remains a monophyletic assemblage, notwithstanding accessions of *G. aridum* from Colima (this also addressed below). 3) The best model of the D-genome donor to allopolyploid (AD-genome cottons), that is, the geographically disjunct *G. raimondii* from Peru [reviewed in {Wendel and Grover 2015}], is well-nested within the subgenus and is phylogenetically sister to the remarkably disjunct Baja California–Galapagos Islands species pair *G. davidsonii* and *G. klotzschianum*; these three species are sister to the Arizona–Sinoloan disjunct species pair *G. thurberi* and *G. trilobum*. 4) The three species from Baja California and adjacent islands, *G. harknessii*, *G. turneri*, and *G. armourianum*, comprise a monophyletic group distinct from the fourth Baja California species *G. davidsonii*, with the first two of these three sister to each other.

The foregoing phylogenetic synopsis evokes a historical biogeography scenario of repeated long-distance dispersals in addition to possible vicariance events that generate geographical disjunctions. It is noteworthy that the aggregate geographical range of the complex extends from southern Arizona to Peru, but with a phylogenetic history that is inconsistent with a single directional radiation across the landscape from any single ancestral home.

Our estimates of initial divergence for the subgenus are consistent with previous estimates from the chloroplast genome (Senchina et al. 2003), and we also find that whereas the subgenus appears to have originated about 6.6 Ma, all surviving species trace to a much more recent origin in the Pleistocene (about 2.5 Ma). Thus, over 4 Myr of evolutionary history of this group is lost, in that no surviving clade traces to the long branch between the D-genome and the remainder of the genus. In addition, nearly all of the biodiversity in the group is more recent in origin, within the last 0.5–2.0 Myr, suggesting a period of both rapid diversification and geographic dispersal extending from Arizona (*G. thurberi*) to the Galapagos Islands (*G. klotzschianum*) and Peru (*G. raimondii*). This temporal framework emphasizes the remarkable and mysterious propensity for long-distance dispersal in the genus *Gossypium*, as reviewed elsewhere (Wendel and Grover 2015).

### Phylogenetic Incongruence and Ancient Hybridization

One of the principal phylogenetic observations of this study is that reconstructions based on nuclear and cpDNA genomes are highly incongruent in a number of respects (fig. 3). Part of the reason for this may be a history of documented (e.g., *G. aridum*, *G. gossypioides*) as well as unobserved interspecific introgression and rapid radiation at the base of the clade, which generates short (i.e., difficult to resolve) internodes. These results recapitulate some of our earlier work (Wendel and Albert 1992; Wendel et al. 1995; Cronn et al. 2003; Cronn and Wendel 2003; Álvarez et al. 2005; Alvarez and Wendel 2006) in which we highlight how comparison between nuclear versus chloroplast phylogenies may inform ancient hybridization events, for such as the evolutionary histories of *G. aridum* and *G. gossypioides*. Populations of the wide-ranging *G. aridum* from the single Mexican state of Colima, for example, share a chloroplast genome with the Baja California–Galapagos Islands species pair *G. davidsonii* and *G. klotzschianum*, whereas populations from the remainder of the range have a chloroplast genome that is phylogenetically included in the rest of the arborescent clade (which includes *Gossypiumlaxum*, *Gossypiumschwendimanii*, and *G. lobatum*) (Wendel and Albert 1992; Alvarez and Wendel 2006). We obtained this same incongruence in our analysis, with the added twist that in the reconstruction based on the nuclear genome, *G. aridum* from Colima appears as the sister to the rest of the arborescent clade, and is thus biphyletic within this group. At present it is unclear whether this position reflects cryptic taxonomic diversity within the group [see discussion in {Wendel and Grover 2015}], or if instead *G. aridum* from Colima was “dragged” to its early-diverging position by nuclear introgression from the *G. davidsonii* and *G. klotzschianum* lineage (with which it share cpDNA genomes). In this respect, we highlight the results from an AFLP survey (Alvarez and Wendel 2006) using a broad sampling of 24 populations of *G. aridum* (including 4 from Colima) as well as the other relevant species, in which it was concluded that the Colima populations are both genetically distinct and contain a comparatively high frequency of AFLP fragments that otherwise are diagnostic of the cpDNA donor clade. Given the biogeographic proximity of Colima to Baja California and hence *G. davidsonii*, we proposed a history, supported here by whole genome (nuclear and chloroplast) sequence data and our dating analysis (fig. 2), of migration of one or more seeds from Baja California to the Colima coast, perhaps during the Pleistocene followed by hybridization and geographically localized nuclear introgression.

Likewise, comparison between the nuclear and chloroplast phylogenies (fig. 3) reveals the previously observed striking incongruence between the nuclear and cpDNA placement of *G. gossypioides*. As described before (Wendel and Albert 1992; Zhao et al. 1998; Cronn et al. 2003; Cronn and Wendel 2003), *G. gossypioides* is recovered as sister to the subgenus *Houzingenia* in nuclear gene trees yet exhibits apparent introgression of repetitive sequences from a different *Gossypium* lineage from Africa. Moreover, and equally extraordinary, this rare species is also confirmed (fig. 3) as sharing a relatively recent cpDNA ancestry with the equally rare Peruvian endemic *G. raimondii* [the only species with which it will form fertile F1 hybrids {Brown and Menzel 1952; Menzel and Brown 1955}]. Thus, *G. gossypioides* likely has undergone two separate instances of introgression: 1) the more recent chloroplast introgression, convincingly shown here for entire chloroplast genomes, and 2) nuclear introgression, as evidenced by the presence of African cotton-like ITS and repetitive DNAs (Wendel et al. 1995; Zhao et al. 1995; Cronn et al. 1996, 2003). This complex genomic history exemplifies how even isolated lineages in different continents (in this case Central America, South America, and Africa) may be linked by a series of remarkable, highly improbable, long-distance dispersal and interspecific hybridization events.

A final comment concerning *G. gossypioides* is that we failed to detect the putative “African” nuclear genomic introgression that is clearly demonstrated by genomic slot blots (Zhao et al. 1998). Although we did not observe introgression using repeat clustering, our analysis does not preclude African-like repeats in the *G. gossypioides* genome. Our results indicate only that this phenomenon is not evident in the present analysis. Analysis of individual clusters fails to reveal any clusters where *G. gossypioides* is significantly different in copy number from the rest of *Houzingenia*. BLAST analysis of the repeats reported by Zhao et al. (1998) suggest the closest cluster is *gypsy* cluster CL31 (72% coverage of AF060607.1); however, this cluster is not enriched in *G. gossypioides* versus the rest of *Houzingenia* (data at https://github.com/IGBB/D_Cottons_USDA; last accessed December 18, 2018). This lack of enrichment is also reflected when the repetitive clones from Zhao et al. (1998) are used to mask each *Houzingenia* genome; that is, neither repetitive clone masks a greater fraction of the *G. gossypioides* genome than any of the other assembled genomes. At present, we cannot explain the different results obtained from these studies, apart from suggesting that the different analytical methods select for different genomic regions or sequence types.

### Molecular Evolutionary Patterns, Processes, and Rates

A primary purpose of this study was to generate genome-wide estimates of molecularly evolutionary patterns, rates, and processes that generate genomic variation. At present, there are few comparable investigations in plants for the time-scale and taxonomic diversity encompassed by this study.

#### Protein Evolution

With respect to genic evolution, we report a relatively narrow range of interspecific nonsynonymous substitution rate (dN), averaging 0.0014 nonsynonymous substitutions per site per million years, with a synonymous substitution rate about an order of magnitude higher (table 3). Thus, evolution at the amino acid level is inferred to be quite slow, averaging only about 1% per codon every 7 Myr. We are unaware of comparable estimates for other plant genera, but we expect that life-history features such as generation time (long in *Gossypium*) will be highly correlated with rates of protein evolution, as they are with rate variation in general (Smith and Donoghue 2008; Gaut et al. 2011). Interestingly, however, indels were estimated to affect as many as one quarter of the gene models in at least one species, with an average of 7% per nonreference species (compared to 1.8% for *G. raimondii* acc. 8 compared with the *G. raimondii*-derived reference genome). Together with the estimates of copy number variability (see Results section), these results warrant a closer inspection on the evolution of genes and gene content in these species.

#### Transposable Elements and the Repetitive Fraction

Similar to previous reports for *Gossypium* (Paterson et al. 2012; Wang et al. 2012; Yu et al. 2012; Li, Fan, et al. 2014; Li et al. 2015; Yuan et al. 2015; Zhang et al. 2015), about half of the genomic space in the species studied here is occupied by transposable elements or their still-similar decaying footprints. As with most flowering plants, a majority of this sequence is due to the prevalence of Class II *gypsy* elements, which comprise about one-third of each of the genomes studied here (fig. 4). Relatively few repetitive elements differ significantly in copy number among the species (fig. 4), indicating a relative genomic stasis in TE content during the last 6.5 Myr, and specifically during the last 2.0 Myr during which most of the modern lineages evolved. In contrast, *gypsy* elements have proliferated in the A-genome diploids (fig. 4) and elsewhere in the genus (Hawkins et al. 2006) following their divergence from the D-genome. We conclude that the TE fraction of the D-genome diploid cotton genomes has been relatively quiescent, especially when compared with other genomes such as those of many grasses, where the repetitive fraction has a far more rapid turnover (Wang and Dooner 2006; Estep et al. 2013; Daron et al. 2014; Luo et al. 2017; Stein et al. 2018). One exception to this generalization is for *G. raimondii* accession 6, in which the *gypsy* element represented by cluster 78 appears to have recently proliferated (supplementary fig. 1, Supplementary Material online). This was a surprising finding, given the exceptionally low levels of nucleotide diversity in this species (Wendel JF, unpublished data) and the small geographic range it occupies in a couple of river valleys in coastal Peru.

Whereas the absolute amount of sequence attributable to *copia* elements is similar among subgenera *Houzingenia*, *Gossypium*, and *Longiloba* (37.4–41.3 Mb, average), this element type represents a larger portion of the genome in *Houzingenia* than in the two larger-genome African subgenera. This observation reflects either a lack of *both copia* element colonization and degradation since divergence of the three subgenera (i.e., stasis of *copia* elements), or convergence of absolute amounts, in a manner that conceals the dynamics of element turnover. Ancestral state reconstructions (images at https://github.com/IGBB/D_Cottons_USDA; last accessed December 18, 2018) suggest that the latter is more likely, as both reduction and increase in copy numbers for the annotated *copia* elements are observed, for both the *Houzingenia* speciesand the African species (represented by *Longiloba*). Whereas *copia* elements comprise a higher proportion of the genome for *Houzingenia* species than for other cottons surveyed (supplementary fig. 5, Supplementary Material online), these elements generally seem to be in decline (table 4), as 65% of accessions experienced a net loss attributable to *copia* elements. This may be due in part to a paradox of TE proliferation; that is, as an element achieves transpositional “success,” the number of homologous regions visible to the recombination-based deletional mechanisms also increases.

**Table 4:** Gain and Loss in *Copia* Elements for Each of the Accessions Clusters

Species	Accession	Numbers of Clusters With Gain Or Loss, per Accession	Sequence Loss in *Copia* Elements (Mb)	Sequence Gain in *Copia* Elements (Mb)
*G. raimondii*	Paterson et al. (2012)	28 ↓ 15 ↑	−7.1	2.4
*G. raimondii*	acc 2	22 ↓ 21 ↑	−7.6	8.6
*G. raimondii*	acc 31	19 ↓ 24 ↑	−6.8	2.4
*G. raimondii*	acc 4	27 ↓ 16 ↑	−10.3	5.6
*G. raimondii*	acc 53	29 ↓ 14 ↑	−11.9	3.4
*G. raimondii*	acc 6	13 ↓ 30 ↑	−1.4	20.2
*G. raimondii*	acc 8	28 ↓ 15 ↑	−7.0	4.9
*G. armourianum*	acc 6	23 ↓ 20 ↑	−4.3	6.1
*G. harknessii*	acc 2	30 ↓ 13 ↑	−9.0	3.9
*G. turneri*	acc 3	20 ↓ 23 ↑	−6.8	6.6
*G. turneri*	acc 7	30 ↓ 13 ↑	−9.2	4.7
*G. turneri*	acc 8	27 ↓ 16 ↑	−7.5	4.2
*G. aridum*	acc 185	23 ↓ 20 ↑	−7.8	5.4
*G. lobatum*	acc 157	26 ↓ 17 ↑	−9.2	5.2
*G. lobatum*	acc 4	20 ↓ 23 ↑	−3.8	6.1
*G. laxum*	acc 4	20 ↓ 23 ↑	−3.1	4.8
*G. schwendimanii*	acc 1	26 ↓ 17 ↑	−5.6	3.4
*G. thurberi*	acc 2	27 ↓ 16 ↑	−5.0	7.0
*G. thurberi*	acc 35	25 ↓ 18 ↑	−5.1	1.2
*G. trilobum*	acc 8	21 ↓ 22 ↑	−2.3	6.5
*G. trilobum*	acc 9	21 ↓ 22 ↑	−5.6	5.8
*G. davidsonii*	acc 27	22 ↓ 21 ↑	−4.8	3.2
*G. klotzschianum*	acc 56	25 ↓ 18 ↑	−7.2	2.5
*G. klotzschianum*	acc 57	24 ↓ 19 ↑	−3.3	5.0
*G. gossypioides*	acc 5	24 ↓ 19 ↑	−5.4	2.0
*G. gossypioides*	acc 7	26 ↓ 17 ↑	−7.9	5.8

#### Genome Differentiation via Insertions and Deletions

Small-scale insertions and deletions are a common form of sequence variation (Sato et al. 2012; Chintalapati et al. 2017; Kapusta et al. 2017; Stein et al. 2018). Despite relatively recent divergence times, we found over one million positions associated with an indel in at least one of the 13 *Houzingenia* species (relative to *Longiloba*), a third of which distinguish one or more *Houzingenia* species. Although indels were found genome-wide, there was variation among chromosomes, which ranged 1.5-fold per Mb. Most indels were small, averaging 6.2 nt, with a range in size of 1–270 nt (table 5). It is likely that some larger indels were missed due to genome sequence incompleteness and because only one species was used as a reference genome.

One notable feature of these data is the observed bias toward deletions over insertions, which averages about 2-fold in number but 2.5- to 5-fold in length (supplementary table 2, Supplementary Material online). The net effect of these dynamics is genome downsizing, with an estimated net loss of about 0.44 Mb per species, with a range between 278 and 555 kb per accession. This observation supports the idea that small deletions may be able to partially counteract historical genome size expansion that originated from TE amplification (Grover and Wendel 2010; Hu et al. 2010; Michael 2014; Simonin and Roddy 2018). Because species in subgenus *Houzingenia* have the smallest genomes in the genus (in which diploids vary about 3-fold in genome size from ∼850 Mb to ∼2,700 Mb), these data suggest that the process of genomic pruning remains active today, or at least it has been in the recent past. Finally, our comparative genomic data reveal, at the finest scale of aligned nucleotides, a dynamic process of genomic downsizing that was inferred from computational modeling a decade ago (Hawkins et al. 2009).

## Supplementary Material

Supplementary data are available at Genome Biology and Evolution online.

## Supplementary Material

Supplementary Data
