# Supplementary material for: Insights into the Evolution of the New World Diploid Cottons (Gossypium, Subgenus Houzingenia) Based on Genome Sequencing
Source: Genome Biol Evol. 2018 Nov 23;11(1):53–71. doi: 10.1093/gbe/evy256 (PMC6320677; doi:10.1093/gbe/evy256)
Supplement: Supplementary Data [file evy256_supp.zip › GroverSupplementaryTables.pdf]

Supplementary Table 1: Number of reads per species/accession and assembly size. E-size is an alternative assembly statistic implemented by the Genome Assembly Gold-standard Evaluations (GAGE) study to evaluate the completeness of the assembled gene space by considering the expected contig size for a randomly selected base.

**Subsection**	**Species**	**Accession**	**# Raw reads**	**Raw coverage (X)**	**# Trimmed reads**	**Trimmed coverage (X)**	**Assembly E-size**
Austroamericana	*G. raimondii*	D5-8	209,266,292	47.6	125,455,638	28.5	2,718
Austroamericana	*G. raimondii*	D5-6	150,875,945	34.3	91,746,782	20.9	1,587
Caducibracteata	*G. armourianum*	D2-1-6	155,005,858	36.2	130,884,124	30.6	3,891
Caducibracteata	*G. harknessii*	JFW	183,115,691	42.8	150,576,667	35.2	5,724
Caducibracteata	*G. turneri*	D10-3	136,351,407	45.0	126,215,258	41.6	29,772
Caducibracteata	*G. turneri*	D10-7	197,515,336	65.1	187,920,299	62.0	45,201
Caducibracteata	*G. turneri*	D10-8	124,131,681	40.9	114,788,764	37.8	33,468
Erioxylum	*G. aridum*	DRD-185	169,506,661	36.9	139,275,121	30.3	4,778
Erioxylum	*G. aridum*	D4-12	158,194,517	34.4	134,374,429	29.2	5,255
Erioxylum	*G. lobatum*	D7-157	173,890,886	41.4	143,417,911	34.1	4,940
Erioxylum	*G. lobatum*	D7-4	169,232,618	36.2	143,485,641	30.7	3,267
Erioxylum	*G. laxum*	D9-4	189,688,054	40.6	166,155,995	35.6	5,530
Erioxylum	*G. schwendimanii*	D11-1	303,290,420	65.3	260,234,325	56.0	10,495
Houzingenia	*G. thurberi*	D1-2	145,820,029	34.7	67,245,582	16.0	---
Houzingenia	*G. thurberi*	D1-35	181,003,659	43.0	157,481,159	37.5	9,074
Houzingenia	*G. trilobum*	D8-8	155,793,983	36.6	130,561,611	30.7	9,368
Houzingenia	*G. trilobum*	D8-9	94,579,207	22.2	81,335,215	19.1	1,765
Integrifolia	*G. davidsonii*	D3D-27	166,777,486	36.7	143,853,161	31.6	10,350
Integrifolia	*G. klotzschianum*	D3K-56	166,667,596	37.9	135,720,623	30.8	5,467
Integrifolia	*G. klotzschianum*	D3K-57	161,773,353	36.8	139,262,455	31.7	7,051
Selera	*G. gossypioides*	D6-5	165,907,958	39.5	142,939,108	34.0	7,621
Selera	*G. gossypioides*	D6-7	167,999,537	40.0	98,890,166	23.5	1,918

Supplementary Table 2: The number and length of indels per accession, and the number of SNPs, relative to *G. longicalyx* (*Longiloba*)

**Number of indels/Mb**		Chr01	Chr02	Chr03	Chr04	Chr05	Chr06	Chr07	Chr08	Chr09	Chr10	Chr11	Chr12	Chr13	allChr
G. raimondii	D5-8	158	131	148	127	98	163	161	169	161	124	134	153	120	141
G. armourianum	D2-1-6	143	132	137	123	99	136	140	153	138	123	121	134	111	130
G. harknessii	D2-2	193	176	189	152	130	198	172	192	177	152	182	201	140	172
G. turneri	D10-7	194	174	188	153	123	200	177	200	177	154	182	199	146	172
G. aridum	D4-185	206	178	207	185	151	203	214	212	201	184	174	190	161	189
G. lobatum	D7-157	212	190	194	187	152	207	208	222	199	188	183	204	171	193
G. laxum	D9-4	169	153	168	148	125	171	172	180	159	151	144	159	139	156
G. schwendimanii	D11-1	231	193	186	190	141	216	219	233	182	194	188	223	161	195
G. thurberi	D1-35	199	163	181	161	126	205	198	207	193	159	174	191	155	177
G. trilobum	D8-8	205	168	191	164	133	210	205	213	199	164	178	201	162	183
G. davidsonii	D3D-27	220	180	209	179	146	229	221	231	209	180	191	221	177	198
G. klotzschianum	D3K-57	220	181	208	179	146	229	220	232	209	180	192	220	177	198
G. gossypioides	D6-5	249	214	234	202	171	250	248	260	233	205	221	246	199	224

**Total insertions and deletions**	Accession	Chr01	Chr02	Chr03	Chr04	Chr05	Chr06	Chr07	Chr08	Chr09	Chr10	Chr11	Chr12	Chr13	allChr (nt)	Total (kb)
G. raimondii	D5-8	-25,262	-21,353	-15,876	-19,203	-13,673	-22,713	-28,094	-27,439	-34,761	-19,942	-22,230	-12,792	-15,108	-278,446	-278
G. armourianum	D2-1-6	-25,064	-22,620	-17,152	-22,034	-18,827	-22,109	-28,855	-27,910	-36,322	-22,941	-24,180	-14,832	-18,272	-301,118	-301
G. harknessii	D2-2	-34,795	-32,626	-26,307	-28,815	-24,828	-33,797	-36,885	-37,497	-47,518	-29,889	-36,067	-23,815	-24,784	-417,623	-418
G. turneri	D10-7	-35,689	-30,350	-26,288	-29,073	-23,284	-32,693	-37,047	-38,776	-46,012	-29,919	-36,102	-22,293	-26,207	-413,733	-414
G. aridum	D4-185	-34,268	-31,313	-26,848	-32,342	-26,149	-32,887	-40,320	-37,537	-49,283	-34,701	-32,278	-19,593	-27,567	-425,086	-425
G. lobatum	D7-157	-34,146	-33,467	-24,837	-32,979	-27,228	-32,182	-38,445	-38,117	-47,935	-33,549	-33,581	-20,229	-26,912	-423,607	-424
G. laxum	D9-4	-28,670	-26,495	-22,894	-25,962	-22,048	-26,100	-33,127	-31,520	-39,339	-28,402	-26,851	-16,811	-22,102	-350,321	-350
G. schwendimanii	D11-1	-38,007	-35,769	-25,017	-31,995	-24,974	-34,593	-40,003	-39,831	-44,377	-35,501	-32,738	-22,594	-27,033	-432,432	-432
G. thurberi	D1-35	-47,918	-37,600	-33,483	-37,344	-28,298	-46,549	-52,835	-52,337	-62,561	-38,946	-43,769	-25,249	-36,203	-543,092	-543
G. trilobum	D8-8	-49,677	-39,087	-33,147	-37,554	-29,361	-46,794	-57,838	-51,790	-63,341	-39,333	-45,898	-25,528	-35,951	-555,299	-555
G. davidsonii	D3D-27	-45,804	-37,810	-31,841	-37,886	-30,325	-44,786	-49,283	-52,588	-59,587	-38,295	-43,470	-27,001	-33,992	-532,668	-533
G. klotzschianum	D3K-57	-45,474	-38,652	-31,468	-37,939	-30,280	-44,807	-49,267	-52,288	-59,261	-38,375	-43,622	-26,779	-33,949	-532,161	-532
G. gossypioides	D6-5	-41,866	-37,695	-30,976	-37,056	-26,859	-38,667	-52,630	-47,663	-58,267	-35,790	-37,207	-26,248	-35,676	-506,600	-507

**Number of insertions**		Chr01	Chr02	Chr03	Chr04	Chr05	Chr06	Chr07	Chr08	Chr09	Chr10	Chr11	Chr12	Chr13	allChr
G. raimondii	D5-8	2,941	2,720	2,338	2,590	2,087	2,849	3,367	3,267	3,967	2,589	2,786	1,895	2,433	35,829
G. armourianum	D2-1-6	2,694	2,792	2,154	2,536	2,110	2,398	2,929	3,027	3,384	2,579	2,539	1,611	2,195	32,948
G. harknessii	D2-2	3,577	3,640	2,909	3,051	2,667	3,379	3,539	3,767	4,393	3,104	3,702	2,358	2,653	42,739
G. turneri	D10-7	3,598	3,635	2,918	3,062	2,585	3,481	3,647	3,886	4,390	3,117	3,714	2,335	2,753	43,121
G. aridum	D4-185	3,867	3,537	3,143	3,677	3,041	3,422	4,409	4,073	4,969	3,699	3,528	2,205	3,087	46,657
G. lobatum	D7-157	3,965	3,722	2,889	3,614	2,992	3,539	4,274	4,309	4,858	3,732	3,688	2,361	3,216	47,159
G. laxum	D9-4	3,129	3,032	2,490	2,889	2,483	2,915	3,562	3,472	3,902	3,018	2,910	1,839	2,625	38,266
G. schwendimanii	D11-1	4,311	3,893	2,827	3,775	2,847	3,632	4,569	4,467	4,509	3,913	3,866	2,612	3,131	48,352
G. thurberi	D1-35	3,550	3,338	2,694	3,211	2,621	3,292	4,036	3,842	4,486	3,291	3,478	2,279	2,901	43,019
G. trilobum	D8-8	3,718	3,471	2,865	3,294	2,824	3,468	4,130	4,015	4,641	3,396	3,568	2,385	3,046	44,821
G. davidsonii	D3D-27	3,948	3,551	3,152	3,527	2,933	3,824	4,428	4,206	4,920	3,557	3,741	2,532	3,278	47,597
G. klotzschianum	D3K-57	3,951	3,558	3,143	3,531	2,934	3,828	4,410	4,227	4,937	3,561	3,756	2,547	3,283	47,666
G. gossypioides	D6-5	4,910	4,639	3,820	4,265	3,765	4,445	5,292	5,182	5,804	4,460	4,827	2,969	3,969	58,347

**Number of deletions**		Chr01	Chr02	Chr03	Chr04	Chr05	Chr06	Chr07	Chr08	Chr09	Chr10	Chr11	Chr12	Chr13	allChr
G. raimondii	D5-8	5,881	5,524	4,424	5,326	4,209	5,452	6,457	6,391	7,412	5,132	5,623	3,543	4,587	69,961
G. armourianum	D2-1-6	5,270	5,480	4,100	5,108	4,258	4,571	5,636	5,715	6,402	5,090	5,072	3,126	4,286	64,114
G. harknessii	D2-2	7,188	7,422	5,749	6,390	5,650	6,743	6,967	7,201	8,130	6,344	7,695	4,765	5,515	85,759
G. turneri	D10-7	7,218	7,276	5,702	6,464	5,332	6,756	7,121	7,533	8,114	6,459	7,678	4,700	5,764	86,117
G. aridum	D4-185	7,630	7,652	6,352	7,838	6,661	6,930	8,664	8,049	9,220	7,758	7,386	4,540	6,301	94,981
G. lobatum	D7-157	7,871	8,227	5,988	7,999	6,734	7,043	8,386	8,393	9,231	7,968	7,764	4,874	6,762	97,240
G. laxum	D9-4	6,293	6,587	5,205	6,302	5,508	5,794	6,932	6,822	7,340	6,364	6,093	3,804	5,495	78,539
G. schwendimanii	D11-1	8,594	8,217	5,698	8,012	6,189	7,401	8,762	8,820	8,347	8,166	7,902	5,295	6,283	97,686
G. thurberi	D1-35	7,557	6,885	5,603	6,780	5,467	7,158	8,048	7,998	9,142	6,595	7,402	4,497	6,164	89,296
G. trilobum	D8-8	7,736	7,045	5,874	6,903	5,703	7,280	8,375	8,145	9,436	6,801	7,584	4,726	6,376	91,984
G. davidsonii	D3D-27	8,355	7,741	6,417	7,608	6,405	7,854	9,038	8,977	9,836	7,622	8,207	5,286	7,073	100,419
G. klotzschianum	D3K-57	8,320	7,782	6,395	7,627	6,413	7,870	9,012	9,014	9,811	7,622	8,300	5,250	7,067	100,483
G. gossypioides	D6-5	8,993	8,792	6,880	8,277	7,203	8,300	9,820	9,649	10,641	8,268	9,039	5,755	7,651	109,268

**Insertion lengths**	Accession	Chr01	Chr02	Chr03	Chr04	Chr05	Chr06	Chr07	Chr08	Chr09	Chr10	Chr11	Chr12	Chr13	allChr
G. raimondii	D5-8	6,970	6,554	5,645	6,557	4,998	7,188	7,862	8,053	10,358	5,892	6,553	4,598	5,758	86,986
G. armourianum	D2-1-6	9,192	9,940	7,862	9,182	6,855	8,249	9,660	11,008	11,880	8,171	8,415	5,197	7,317	112,928
G. harknessii	D2-2	12,555	12,211	10,515	10,789	9,348	12,013	11,815	13,010	14,921	9,944	13,336	8,266	9,638	148,361
G. turneri	D10-7	13,345	13,753	10,909	10,912	9,082	12,834	12,688	14,040	15,865	10,275	14,172	8,545	10,184	156,604
G. aridum	D4-185	13,002	12,367	10,791	13,157	9,965	11,724	14,657	14,395	16,911	12,734	12,188	7,240	9,901	159,032
G. lobatum	D7-157	13,909	13,355	10,159	12,851	9,894	12,268	14,029	15,464	16,486	12,799	12,933	8,106	10,749	163,002
G. laxum	D9-4	9,772	10,560	8,166	9,963	8,003	10,118	11,479	12,074	13,341	9,677	9,390	5,495	8,996	127,034
G. schwendimanii	D11-1	16,098	14,050	9,794	14,664	9,652	13,116	16,792	17,404	16,102	13,958	14,477	9,014	10,970	176,091
G. thurberi	D1-35	11,826	11,213	8,668	11,294	8,765	11,516	13,267	13,064	15,202	10,769	11,740	7,690	9,858	144,872
G. trilobum	D8-8	12,310	11,502	9,849	11,340	9,404	12,296	13,506	13,757	16,027	11,751	11,482	8,222	10,104	151,550
G. davidsonii	D3D-27	13,736	11,433	10,842	11,587	9,560	13,115	15,219	13,987	16,999	11,924	12,854	8,170	11,727	161,153
G. klotzschianum	D3K-57	13,574	11,244	10,844	11,628	9,616	13,062	15,381	14,251	17,421	12,163	12,991	8,184	11,621	161,980
G. gossypioides	D6-5	21,001	19,376	16,102	18,317	17,042	20,145	21,632	23,519	26,526	18,523	22,048	12,156	16,757	253,144

**Deletion lengths**	Accession	Chr01	Chr02	Chr03	Chr04	Chr05	Chr06	Chr07	Chr08	Chr09	Chr10	Chr11	Chr12	Chr13	allChr
G. raimondii	D5-8	-32,232	-27,907	-21,521	-25,760	-18,671	-29,901	-35,956	-35,492	-45,119	-25,834	-28,783	-17,390	-20,866	-365,432
G. armourianum	D2-1-6	-34,256	-32,560	-25,014	-31,216	-25,682	-30,358	-38,515	-38,918	-48,202	-31,112	-32,595	-20,029	-25,589	-414,046
G. harknessii	D2-2	-47,350	-44,837	-36,822	-39,604	-34,176	-45,810	-48,700	-50,507	-62,439	-39,833	-49,403	-32,081	-34,422	-565,984
G. turneri	D10-7	-49,034	-44,103	-37,197	-39,985	-32,366	-45,527	-49,735	-52,816	-61,877	-40,194	-50,274	-30,838	-36,391	-570,337
G. aridum	D4-185	-47,270	-43,680	-37,639	-45,499	-36,114	-44,611	-54,977	-51,932	-66,194	-47,435	-44,466	-26,833	-37,468	-584,118
G. lobatum	D7-157	-48,055	-46,822	-34,996	-45,830	-37,122	-44,450	-52,474	-53,581	-64,421	-46,348	-46,514	-28,335	-37,661	-586,609
G. laxum	D9-4	-38,442	-37,055	-31,060	-35,925	-30,051	-36,218	-44,606	-43,594	-52,680	-38,079	-36,241	-22,306	-31,098	-477,355
G. schwendimanii	D11-1	-54,105	-49,819	-34,811	-46,659	-34,626	-47,709	-56,795	-57,235	-60,479	-49,459	-47,215	-31,608	-38,003	-608,523
G. thurberi	D1-35	-59,744	-48,813	-42,151	-48,638	-37,063	-58,065	-66,102	-65,401	-77,763	-49,715	-55,509	-32,939	-46,061	-687,964
G. trilobum	D8-8	-61,987	-50,589	-42,996	-48,894	-38,765	-59,090	-71,344	-65,547	-79,368	-51,084	-57,380	-33,750	-46,055	-706,849
G. davidsonii	D3D-27	-59,540	-49,243	-42,683	-49,473	-39,885	-57,901	-64,502	-66,575	-76,586	-50,219	-56,324	-35,171	-45,719	-693,821
G. klotzschianum	D3K-57	-59,048	-49,896	-42,312	-49,567	-39,896	-57,869	-64,648	-66,539	-76,682	-50,538	-56,613	-34,963	-45,570	-694,141
G. gossypioides	D6-5	-62,867	-57,071	-47,078	-55,373	-43,901	-58,812	-74,262	-71,182	-84,793	-54,313	-59,255	-38,404	-52,433	-759,744

**Total Indels vs Longiloba**	Accession	Chr01	Chr02	Chr03	Chr04	Chr05	Chr06	Chr07	Chr08	Chr09	Chr10	Chr11	Chr12	Chr13	allChr
G. raimondii	D5-8	38,016	35,366	29,007	33,907	29,542	34,395	40,179	39,113	43,769	33,738	37,798	24,447	32,436	451,713
G. armourianum	D2-1-6	37,158	35,394	28,499	33,635	29,614	33,063	38,920	38,197	42,176	33,686	37,000	23,746	31,897	442,985
G. harknessii	D2-2	39,959	38,184	30,903	35,432	31,563	36,216	40,861	40,423	44,913	35,465	40,786	26,132	33,584	474,421
G. turneri	D10-7	40,010	38,033	30,865	35,517	31,163	36,331	41,123	40,874	44,894	35,593	40,781	26,044	33,933	475,161
G. aridum	D4-185	40,691	38,311	31,740	37,506	32,948	36,446	43,428	41,577	46,579	37,474	40,303	25,754	34,804	487,561
G. lobatum	D7-157	41,030	39,071	31,122	37,604	32,972	36,676	43,015	42,157	46,479	37,717	40,841	26,244	35,394	490,322
G. laxum	D9-4	38,616	36,741	29,940	35,182	31,237	34,803	40,849	39,749	43,632	35,399	38,392	24,652	33,536	462,728
G. schwendimanii	D11-1	42,099	39,232	30,770	37,778	32,282	37,127	43,686	42,742	45,246	38,096	41,157	26,916	34,830	491,961
G. thurberi	D1-35	40,301	37,345	30,542	35,982	31,334	36,544	42,439	41,295	46,018	35,903	40,269	25,785	34,481	478,238
G. trilobum	D8-8	40,648	37,638	30,984	36,188	31,773	36,842	42,860	41,615	46,467	36,214	40,541	26,120	34,838	482,728
G. davidsonii	D3D-27	41,497	38,414	31,814	37,126	32,584	37,772	43,821	42,638	47,146	37,196	41,337	26,827	35,767	493,939
G. klotzschianum	D3K-57	41,465	38,462	31,783	37,149	32,593	37,792	43,777	42,696	47,138	37,200	41,445	26,806	35,766	494,072
G. gossypioides	D6-5	43,097	40,553	32,945	38,533	34,214	38,839	45,467	44,286	48,835	38,745	43,255	27,733	37,036	513,538

**Total SNPs vs Longiloba**	Accession	Chr01	Chr02	Chr03	Chr04	Chr05	Chr06	Chr07	Chr08	Chr09	Chr10	Chr11	Chr12	Chr13	all Chr
G. raimondii	D5-8	606,538	677,600	474,136	685,713	688,476	528,471	644,967	604,760	730,018	654,029	638,891	394,752	######	7,909,366
G. armourianum	D2-1-6	575,078	657,872	455,524	659,199	663,111	492,335	602,225	574,089	689,900	632,322	603,551	369,121	######	7,525,371
G. harknessii	D2-2	627,136	718,843	497,218	697,164	722,347	544,739	637,650	613,087	740,712	674,374	671,507	411,030	######	8,140,633
G. turneri	D10-7	626,097	716,710	497,928	700,517	712,610	546,083	642,375	622,165	741,527	676,104	672,285	409,005	######	8,155,064
G. aridum	D4-185	646,216	734,980	524,144	758,891	764,551	554,751	696,237	647,753	783,306	730,032	680,316	413,024	######	8,555,662
G. lobatum	D7-157	658,229	751,930	517,781	759,791	767,490	567,277	692,299	659,187	788,974	736,793	694,432	421,936	######	8,651,866
G. laxum	D9-4	607,810	696,652	487,897	700,687	714,134	526,646	643,065	611,046	727,036	678,741	640,724	389,673	######	8,015,127
G. schwendimanii	D11-1	669,362	750,989	505,088	761,128	746,012	569,425	696,690	660,440	766,248	737,629	695,092	429,550	######	8,606,096
G. thurberi	D1-35	624,058	700,390	488,753	709,191	714,260	540,316	657,252	618,908	741,817	681,589	659,731	403,909	######	8,139,420
G. trilobum	D8-8	630,824	707,541	495,729	715,223	725,698	545,367	665,106	626,584	750,650	688,389	666,859	408,382	######	8,232,774
G. davidsonii	D3D-27	655,423	731,643	516,053	742,395	751,512	568,085	689,699	650,363	778,405	716,287	687,369	423,400	######	8,539,202
G. klotzschianum	D3K-57	655,240	733,305	515,591	743,358	751,842	568,113	689,413	651,137	778,903	715,835	689,482	423,238	######	8,545,127
G. gossypioides	D6-5	640,512	719,426	501,879	723,068	737,597	555,059	674,562	635,847	761,366	700,119	678,852	415,326	######	8,359,287

**SNPs:Indels**	Accession	Chr01	Chr02	Chr03	Chr04	Chr05	Chr06	Chr07	Chr08	Chr09	Chr10	Chr11	Chr12	Chr13	allChr
G. raimondii	D5-8	16.0	19.2	16.3	20.2	23.3	15.4	16.1	15.5	16.7	19.4	16.9	16.1	17.9	17.5
G. armourianum	D2-1-6	15.5	18.6	16.0	19.6	22.4	14.9	15.5	15.0	16.4	18.8	16.3	15.5	17.3	17.0
G. harknessii	D2-2	15.7	18.8	16.1	19.7	22.9	15.0	15.6	15.2	16.5	19.0	16.5	15.7	17.4	17.2
G. turneri	D10-7	15.6	18.8	16.1	19.7	22.9	15.0	15.6	15.2	16.5	19.0	16.5	15.7	17.4	17.2
G. aridum	D4-185	15.9	19.2	16.5	20.2	23.2	15.2	16.0	15.6	16.8	19.5	16.9	16.0	17.9	17.5
G. lobatum	D7-157	16.0	19.2	16.6	20.2	23.3	15.5	16.1	15.6	17.0	19.5	17.0	16.1	18.0	17.6
G. laxum	D9-4	15.7	19.0	16.3	19.9	22.9	15.1	15.7	15.4	16.7	19.2	16.7	15.8	17.6	17.3
G. schwendimanii	D11-1	15.9	19.1	16.4	20.1	23.1	15.3	15.9	15.5	16.9	19.4	16.9	16.0	17.8	17.5
G. thurberi	D1-35	15.5	18.8	16.0	19.7	22.8	14.8	15.5	15.0	16.1	19.0	16.4	15.7	17.4	17.0
G. trilobum	D8-8	15.5	18.8	16.0	19.8	22.8	14.8	15.5	15.1	16.2	19.0	16.4	15.6	17.4	17.1
G. davidsonii	D3D-27	15.8	19.0	16.2	20.0	23.1	15.0	15.7	15.3	16.5	19.3	16.6	15.8	17.6	17.3
G. klotzschianum	D3K-57	15.8	19.1	16.2	20.0	23.1	15.0	15.7	15.3	16.5	19.2	16.6	15.8	17.6	17.3
G. gossypioides	D6-5	14.9	17.7	15.2	18.8	21.6	14.3	14.8	14.4	15.6	18.1	15.7	15.0	16.6	16.3

Supplementary Table 3: Accessions used in the present study

Subsection	Species	Accessio n	Sequenced
Caducibracteata	*G. armourianum*	D2-1-6	BGI
Erioxylum	*G. aridum*	D4-12	BGI
Erioxylum	*G. lobatum*	D7-4	BGI
Erioxylum	*G. laxum*	D9-4	BGI
Erioxylum	*G. schwendimanii*	D11-1	BGI
Houzingenia	*G. thurberi*	D1-35	BGI
Houzingenia	*G. trilobum*	D8-8	BGI
Integrifolia	*G. davidsonii*	D3D-27	BGI
Integrifolia	*G. klotzschianum*	D3K-57	BGI
Selera	*G. gossypioides*	D6-5	BGI
Caducibracteata	*G. turneri*	D10-3	Novogene
Caducibracteata	*G. turneri*	D10-7	Novogene
Caducibracteata	*G. turneri*	D10-8	Novogene
Austroamericana	*G. raimondii*	D5-8	NXT
Austroamericana	*G. raimondii*	D5-6	NXT
Houzingenia	*G. thurberi*	D1-2	NXT
Houzingenia	*G. trilobum*	D8-9	NXT
Selera	*G. gossypioides*	D6-7	NXT
Integrifolia	*G. klotzschianum*	D3K-56	USDA
Caducibracteata	*G. harknessii*	JFW	USDA
Erioxylum	*G. aridum*	DRD-185	USDA
Erioxylum	*G. lobatum*	D7-157	USDA

BGI: Samples submitted to BGI Genomics (Hong Kong) for Illumina library preparation and 2x100bp sequencing.
NXT: Samples prepared in-house at the USDA-ARS GBRU core facility using Nextera and sequenced as 2x100 bp.
USDA: Samples prepared at the USDA-ARS GBRU core facility using Accel-NGS 2S PCR-Free (Product number 20024 with adapter set 26396, Swift Biosciences, Ann Arbor, MI, USA) and
Novogene: Samples submitted to Novogene (Beijing) for Illumina library preparation and 2x150bp sequencing.
