# Supplementary figures and images for: Insights into the Evolution of the New World Diploid Cottons (Gossypium, Subgenus Houzingenia) Based on Genome Sequencing
Source: Genome Biol Evol. 2018 Nov 23;11(1):53–71. doi: 10.1093/gbe/evy256 (PMC6320677; doi:10.1093/gbe/evy256)

Supplementary Figure 1

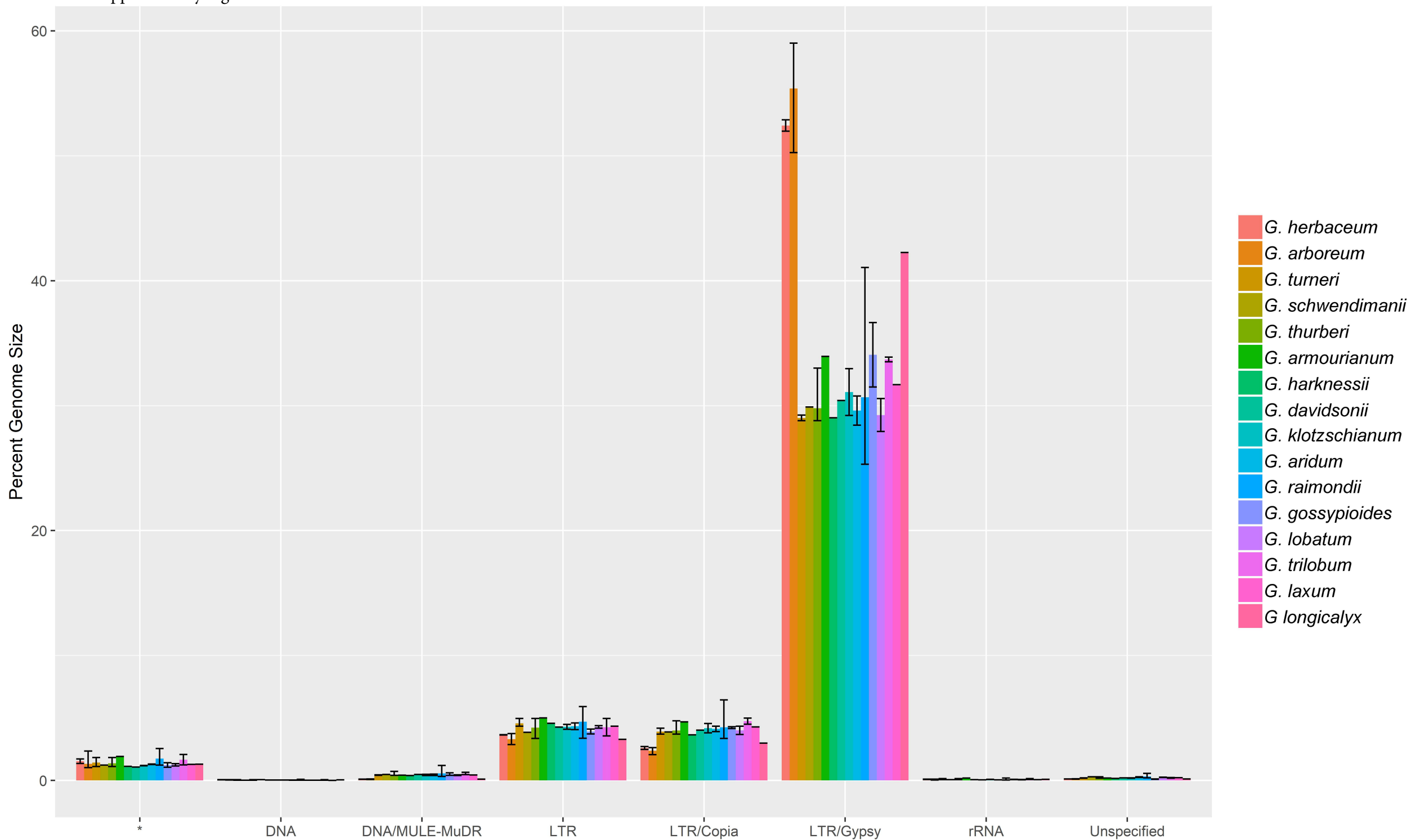

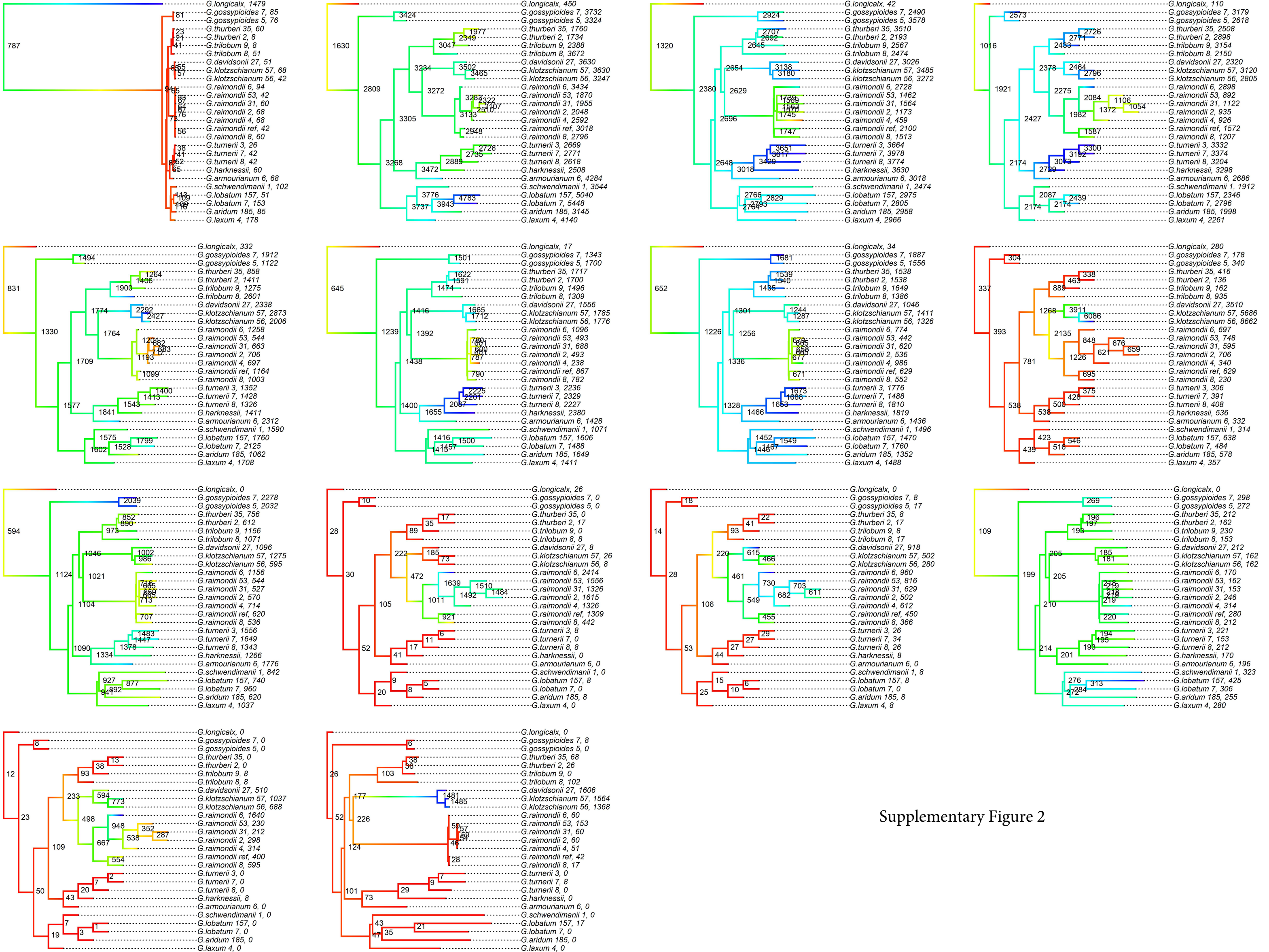

Supplementary Figure 2

Supplementary Figure 3

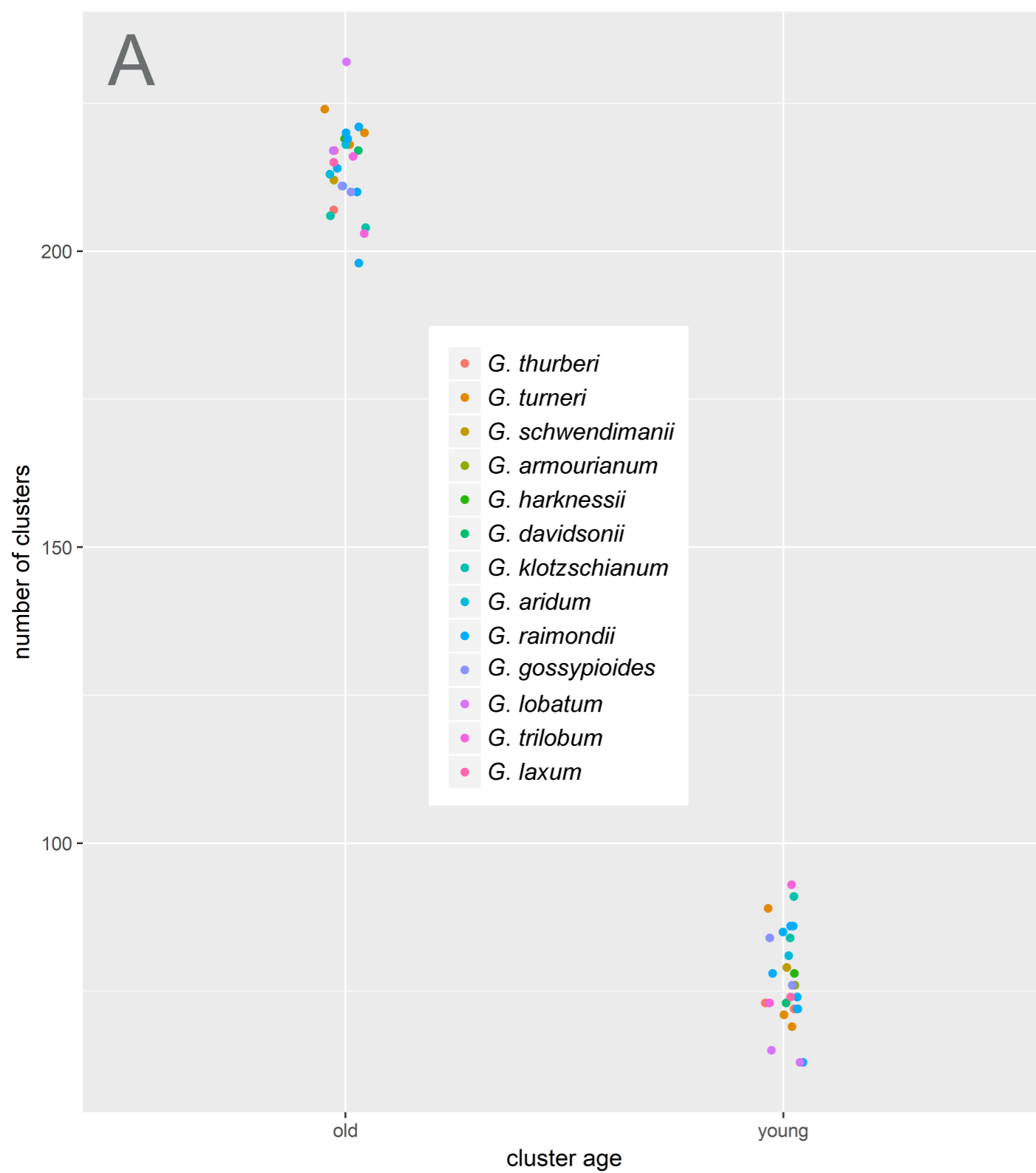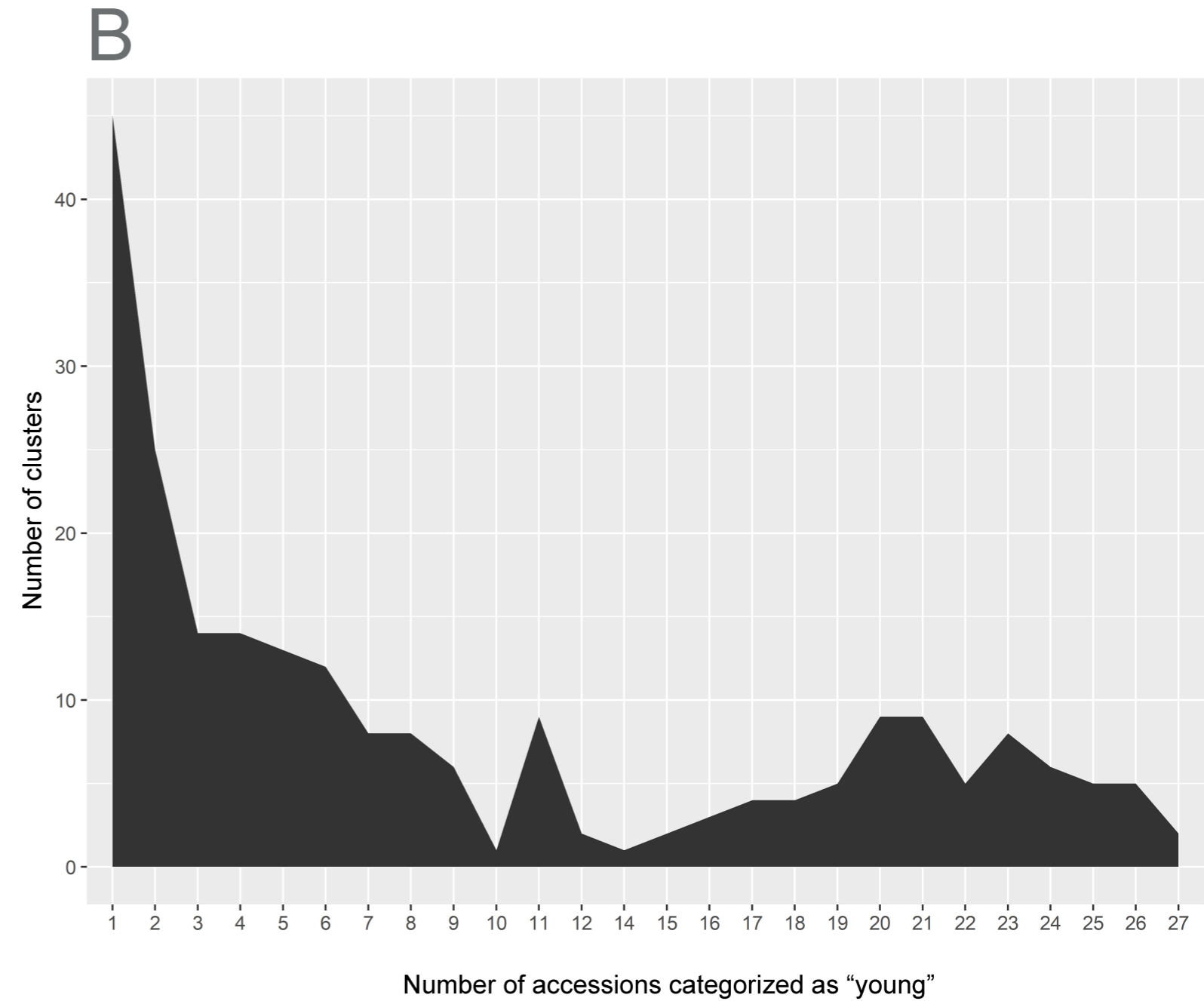

Supplementary Figure 4

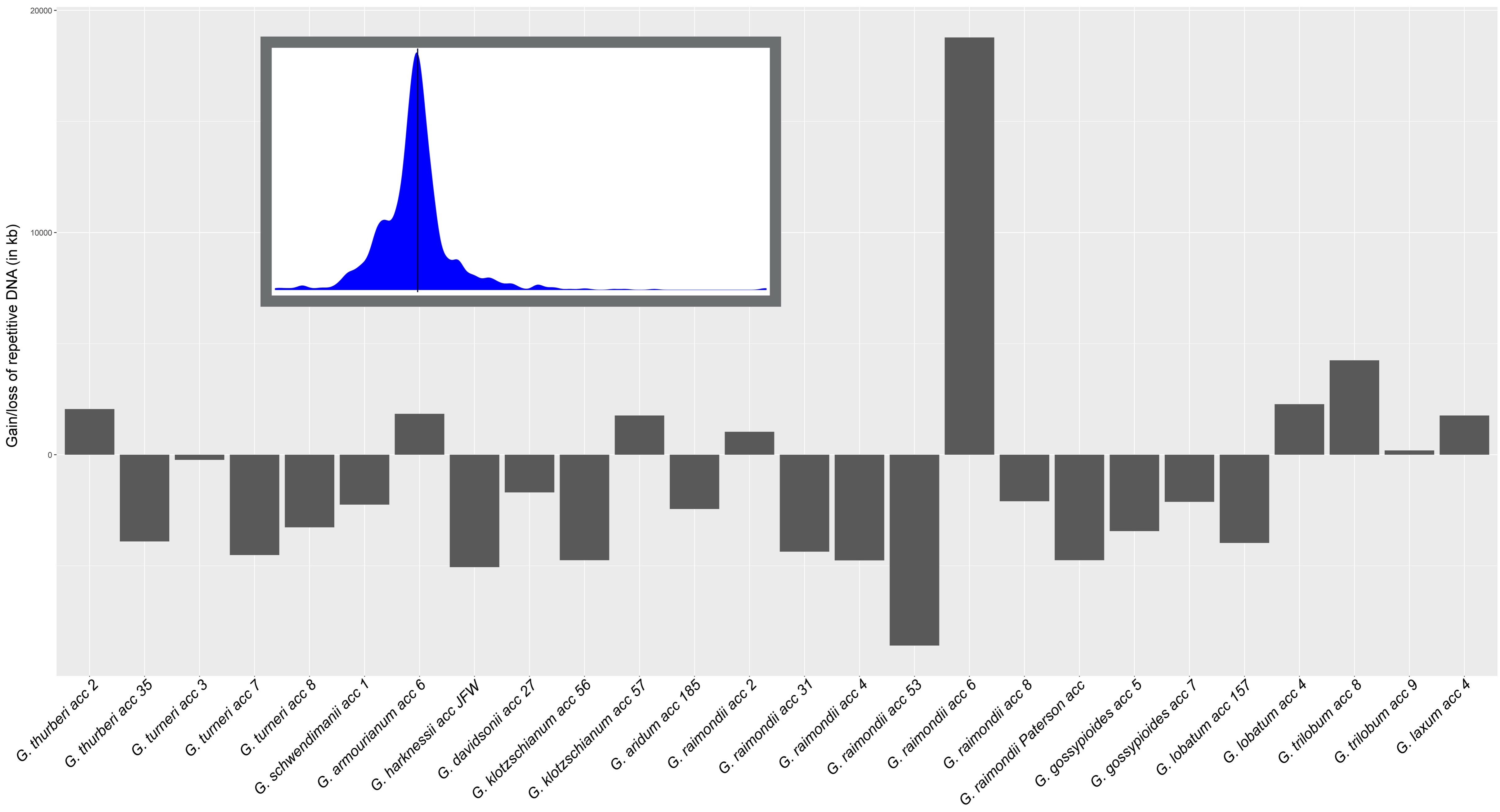

Supplement: Supplementary Data [file evy256_supp.zip › GroverSupplementaryFigures.pdf]
